# Breast Cancer Risk in over 1.3 Million Women on Antipsychotic Therapy: Life-Saving Drugs or Hidden Trigger for Breast Cancer?

**DOI:** 10.3390/medsci14020205

**Published:** 2026-04-20

**Authors:** Enrico Altiero Giusto, Vittorio Oteri, Giorgio Guido, Delia Anamaria Bogdan, Jacopo Giuliani, Carlotta Giorgi, Paolo Pinton, Francesco Fiorica

**Affiliations:** 1 Department of Medical Sciences, Section of Experimental Medicine and Laboratory of Technologies for Advanced Therapy (LTTA), University of Ferrara, 44121 Ferrara, Italy; 2 Azienda Ospedaliero-Universitaria S. Anna, Via Aldo Moro 8, 44124 Ferrara, Italy; 3 Department of Clinical and Experimental Medicine, Endocrinology Section, Garibaldi-Nesima Hospital, University of Catania, Via Palermo, 636, 95122 Catania, Italy; 4 Department of Biomedical and Biotechnological Sciences, University of Catania, Via S. Sofia 89, 95123 Catania, Italy; 5 Department of Sciences, Sapienza University of Rome, Piazzale Aldo Moro 5, 00185 Rome, Italy; 6 Department of Clinical Oncology, Section of Radiation Oncology and Nuclear Medicine, AULSS 9 Scaligera, 37122 Verona, Italy; 7 Biomedical Research Center, Kansai Medical University, 10-15, Fumizono-cho Moriguchi-shi, Osaka 570-8507, Japan; 8 Maria Cecilia Hospital, GVM Care & Research, Via Corriera 1, 48033 Cotignola, Italy; 9 Department of Clinical Oncology, Section of Medical Oncology, AULSS 9 Scaligera, 37122 Verona, Italy

**Keywords:** breast cancer, neuroleptics, antipsychotic, drug therapy

## Abstract

**Introduction:** Antipsychotic (AP) medications are widely prescribed beyond psychotic disorders, yet their long-term safety profile regarding breast cancer (BC) risk remains uncertain. **Methods:** We conducted a systematic review and meta-analysis of observational studies evaluating the association between AP exposure and incident BC. Eligible studies reported adjusted odds ratios (ORs) with 95% confidence intervals for any AP, prolactin-increasing antipsychotics (PIAPs), or prolactin-sparing antipsychotics (PSAPs). Study quality was assessed using the modified Newcastle-Ottawa Scale (mNOS), and certainty of evidence was graded with the GRADE framework. Random-effect models were used to pool effect estimates by exposure category, duration, and cumulative Defined Daily Dose (DDD). **Results:** Nine high-quality observational studies encompassing 108 effect estimates were included. Most studies achieved mNOS scores of 9, yet GRADE certainty ranged from very low to moderate, with the overall body of evidence graded as low certainty due primarily to residual confounding. Any AP exposure was associated with a modestly increased BC risk, particularly with long-term use: use for >5 years yielded pooled ORs around 1.5–1.6, while short-to-medium duration (1–5 years) showed smaller increases (pooled ORs in the range 1.2–1.3). For PIAPs, both longer duration (>5 years) and higher cumulative exposure (>1000–2000 DDDs) were consistently associated with ORs/HRs in the 1.3–1.6 range, suggesting a possible dose–response pattern. Histological analyses indicated stronger associations for ductal than lobular BC, and elevated risks were observed across age strata, including women aged <55 and ≥70 years. **Discussion:** This meta-analysis suggests that chronic exposure to prolactin-increasing antipsychotics is associated with a potentially clinically relevant increase in BC risk, whereas prolactin-sparing agents do not show a clear signal of harm. However, the certainty of this association is limited by inconsistently measured confounders and by the observational nature of the data. These findings support a cautious, individualized approach in which clinicians preferentially consider PSAPs when appropriate, discuss BC risk as part of shared decision-making, and integrate tailored screening strategies for women requiring long-term PIAP therapy. Further high-quality pharmacoepidemiologic studies with better confounder control and mechanistic integration are needed to refine risk estimates and inform preventive neuropsychopharmacology.

## 1. Introduction

The clinical application of antipsychotic (AP) medications has expanded considerably in recent years. Beyond their established roles in treating schizophrenia and bipolar disorders, these agents are now frequently prescribed off-label for mood stabilization, refractory anxiety, and sleep disturbances [1,2]. While their psychiatric efficacy is well-documented, the long-term effects of APs on female oncological health, particularly regarding breast cancer (BC) risk, remain under active scientific investigation.

This concern arises from the dopaminergic mechanism of action of most APs, which function as D2 receptor antagonists and frequently induce significant hyperprolactinemia. Since prolactin serves as a potent mitogen for mammary epithelial cells [3], the hypothesis that chronic hormonal stimulation may promote tumorigenesis is gaining traction. Nevertheless, the literature remains inconclusive; some observational studies report a clear correlation, whereas others do not demonstrate statistically significant associations. This inconsistency has led to the absence of unified guidelines for risk management.

This meta-analysis was designed to systematically evaluate the existing evidence by analyzing a cohort of over 1.3 million participants. The analysis specifically examines the impact of treatment duration, cumulative dosage (defined daily doses, DDDs), and pharmacological class, with a distinction between prolactin-increasing (PIAPs) and prolactin-sparing (PSAPs) agents. Assessing the interaction between pharmacological variables and histological subtypes is critical for optimizing patient care. The findings seek to bridge the gap between psychiatric efficacy and oncological safety, supporting the development of preventive neuropsychopharmacology models in which screening protocols are tailored to individual medication histories.

## 2. Materials and Methods

We followed a standardized procedure and reported the results in accordance with the Preferred Reporting Items for Systematic Reviews and Meta-Analyses (PRISMA) guidelines [4,5] and Meta-analyses of Observational Studies in Epidemiology (MOOSE) guidelines [6,7]. The meta-analysis is recorded in PROSPERO n. CRD420251169808.

### 2.1. Search Strategy

**Figure 1:**
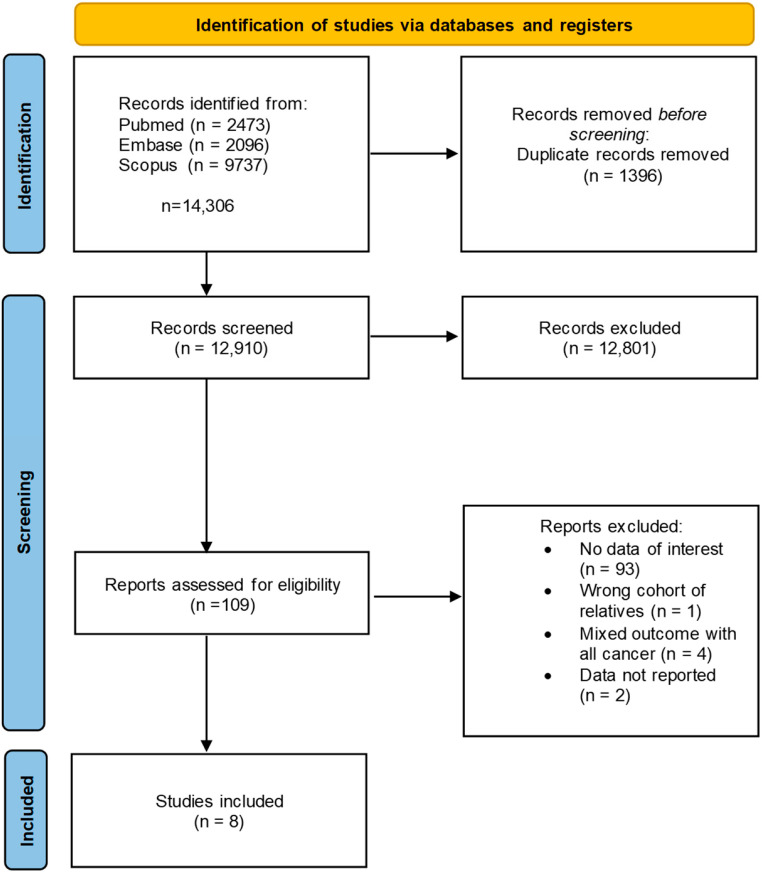
PRISMA flowchart selection progress.

Eligible studies were identified through systematic screening of PubMed, Embase, and Scopus from database inception to 13 June 2025. The search strategy incorporated MeSH descriptors for neuroleptics and breast neoplasms, restricting results to human studies. To maintain currency, an updated search was performed on 16 March 2026, using the original search strings. Electronic retrieval was complemented by manual review of reference lists from all included articles and relevant systematic reviews (see Figure 1). This snowballing approach enabled the identification of citations potentially missed by database indexing. Detailed search protocols are available in the Supplementary Materials.

### 2.2. Eligibility Criteria and Study Selection

Eligibility screening was independently conducted by two investigators (E.A.G. and P.P.) in a blinded manner. Discrepancies were resolved by consensus or, if necessary, by a third senior reviewer (F.F.). Selection criteria were not restricted by geography, sex, or ethnicity. Studies were included if they met the following parameters:

1. Design: Randomized controlled trials (RCTs), population-based case–control, nested case–control, or prospective/retrospective cohort studies.
2. Outcome: The primary endpoint was the incidence of breast cancer.
3. Statistical Reporting: Availability of Odds Ratios (ORs), Relative Risks (RRs), or Hazard Ratios (HRs) with corresponding 95% Confidence Intervals (CIs), or sufficient raw data for their derivation.
4. Exposure Definition: AP exposure was characterized by at least one documented prescription or dispense recorded in validated national pharmacy databases or confirmed via healthcare professional interviews within a 14-day window.

Studies focused solely on mortality or review-only manuscripts were excluded. To prevent data inflation, duplicate cohorts were identified, and only the most comprehensive publication, defined as the one with the longest follow-up, was retained for analysis.

### 2.3. Data Extraction and Quality Assessment

A standardized data extraction protocol was implemented independently by two authors (E.A.G. and C.G.), with F.F. serving as an arbitrator. Extracted data included cohort size, publication year, follow-up duration, exposure confirmation methodology, pharmacological class, and geographical location. Effect estimates (ORs, RRs, or HRs) adjusted for the most comprehensive set of confounding variables in the primary studies were prioritized to maximize precision.

Methodological quality was rigorously assessed using the 9-star modified Newcastle-Ottawa Scale [8,9] (mNOS), covering three key domains: cohort selection, group comparability, and outcome/exposure verification. Studies attaining a full 9-star score were classified as high-quality. The certainty of evidence was further evaluated using the GRADE [10] framework.

### 2.4. Anatomical Therapeutic Chemical Classification

Antipsychotic exposure in this study was operationalised at three hierarchical levels: any antipsychotic, prolactin-increasing antipsychotics (PIAPs), and prolactin-sparing antipsychotics (PSAPs), all classified within the N05A group of the WHO Anatomical Therapeutic Chemical (ATC) system. Any antipsychotic corresponds to the entire N05A category, which includes both typical (first-generation) and atypical (second-generation) neuroleptics used for schizophrenia, bipolar disorder, and related indications. Within this overarching class, PIAPs were defined as antipsychotic agents within N05A that are known to induce sustained hyperprolactinemia through strong D2 receptor blockade in the tuberoinfundibular pathway (predominantly high-potency typicals and some atypicals), whereas PSAPs were defined as N05A agents with a prolactin-sparing profile, characterized by minimal, transient, or clinically negligible prolactin elevation, such as several second-generation antipsychotics with lower D2 occupancy or more rapid dissociation kinetics. For meta-analytic purposes, individual N05A substances from these subclasses were aggregated into three pharmacological exposure groups, any antipsychotic (N05A overall), PIAPs (N05A with a prolactin-increasing profile), and PSAPs (N05A with a prolactin-sparing profile), to align ATC classification with the mechanistic hypothesis of prolactin-mediated breast carcinogenesis.

Specifically:

- All antipsychotics are coded within N05A and its subclasses (e.g., N05AA phenothiazines, N05AD butyrophenones, N05AH diazepines/oxazepines/thiazepines, N05AL benzamides, N05AX other antipsychotics).
- Drugs typically considered prolactin-increasing (PIAPs), such as many first-generation antipsychotics and agents like risperidone or amisulpride, have their own N05A-subgroup codes (e.g., haloperidol N05AD01, risperidone N05AX08, amisulpride N05AL05).
- Drugs commonly regarded as prolactin-sparing (PSAPs), for example aripiprazole (N05AX12), quetiapine (N05AH04), ziprasidone (N05AE04), clozapine (N05AH02).

### 2.5. Statistical Analysis

Random-effect models were utilized to synthesize pooled estimates, accounting for anticipated inter-study heterogeneity [11]. When 95% confidence intervals (CIs) were unavailable, they were derived under the assumption of a Gaussian distribution. Primary estimates underwent log-transformation to ensure symmetry before back-transformation for reporting. Between-study variance was estimated using the restricted maximum likelihood (REML) method [12,13,14].

Statistical analyses were conducted using R (v2024; R Foundation for Statistical Computing). Heterogeneity, precision, and publication bias were evaluated using Galbraith and Funnel plots [15]. The robustness of the estimates was confirmed at the 0.05 alpha level. Each comprehensive forest plot represents an independent meta-analytic synthesis.

### 2.6. Ethics Statement

This comprehensive meta-analysis was managed by analyzing data from published manuscripts. The eligible studies had obtained the ethical agreement from their corresponding Institutional Review Boards, as recognized in the original papers.

## 3. Results

### 3.1. Literature Search

The literature search generated 108 different effect estimates in 8 observational studies comprising 1,371,985 participants. The publication dates ranged from 1978 to 2022.

**Figure 2:**
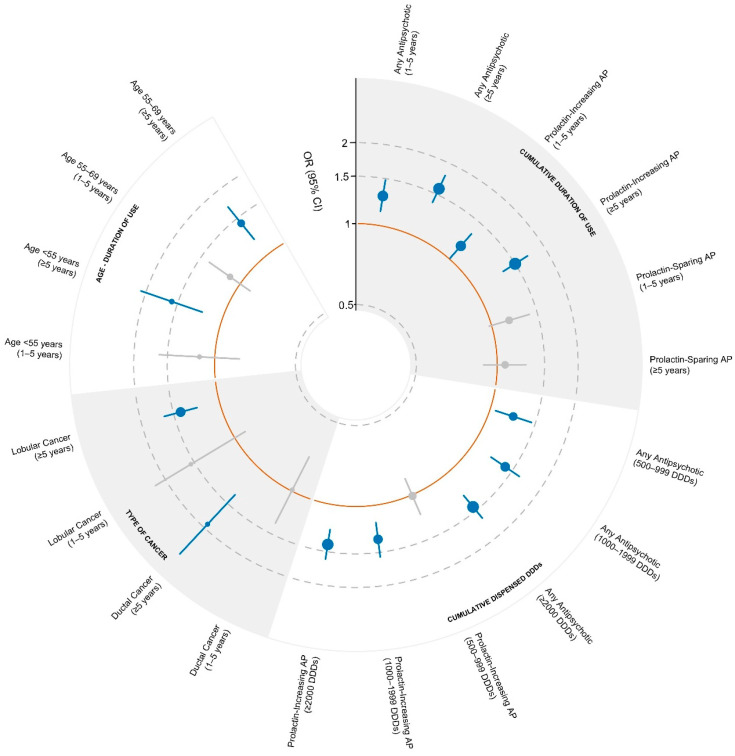
Summary of evidence. The pooled OR associated with breast cancer for each patient condition, duration, histotypes and demographic characteristic. The horizontal lines through the circles represent the 95% confidence interval (C.I.) for each study statistically significant result are coloured in blue, while not significant are coloured grey; each different variable corresponds to one meta-analysis. Results are reported in logarithmic scale for symmetry.

A total of 25 meta-analytic pooled estimates were appraised for the main analysis: 12 (48%) reported effects that were significant at *p* < 0.001, two (8%) at *p* = 0.01 and one (4%) at *p* = 0.03 (See Table 1 and Figure 2).

### 3.2. Quality Assessment of the Studies

The Modified Newcastle-Ottawa Scale rating for all studies was high: five studies received 9 stars (67%), two studies received 8 stars (25%), and one study received 7 stars (8%) (see Supplementary Materials Table S3). Despite the high NOS scores, the GRADE assessment highlighted a more nuanced landscape, with the certainty of evidence ranging from very low to moderate. Across the included large observational studies, the GRADE assessment indicates low overall certainty that long-term or prolactin-elevating antipsychotic use modestly increases breast cancer risk. The main limitation is residual confounding, because even the registry cohorts cannot fully adjust for key reproductive and lifestyle factors such as parity, age at first birth, family history, alcohol, smoking, and obesity in a consistent way; this leads to a downgrade for risk of bias. Nevertheless, effect estimates are reasonably precise and broadly consistent: most high-quality studies report adjusted relative risks or odds ratios in the 1.2–1.6 range for high cumulative or long-duration exposure to prolactin-increasing agents while low or short-term exposure is often compatible with no important increase. Translating this into absolute terms, for a typical baseline postmenopausal breast cancer risk of about 3–4% over 10–15 years [24], a 30–50% relative increase would correspond to an absolute excess of roughly 1–2 additional cases per 100 women with long-term prolactin-elevating antipsychotic exposure, but because of the low GRADE certainty, this estimate should be interpreted cautiously and framed as an approximate range rather than a precise risk.

### 3.3. Pharmacological Exposure and BC Risk

#### 3.3.1. Any Antipsychotic

Exposure to any AP was positively correlated with BC incidence. Short-to-medium term use (1–5 years) yielded an OR of 1.29 (95% CI: 1.13–1.48). Notably, long-term exposure (>5 years) significantly escalated the risk to an OR of 1.58 (95% CI: 1.39–1.79), invasive BC is reported with an HR 1.13, CI 95% 0.6–2.11; a clear dose–response relationship was observed: 500–999 Defined Daily Doses (DDDs, a standard measure of drug consumption), OR 1.23; 1000–1999 DDDs, OR 1.40; and ≥2000 DDDs, OR 1.44 (95% CI: 1.27–1.63).

#### 3.3.2. Prolactin-Increasing Antipsychotic

The risk associated with PIAPs is also associated with a higher risk of BC.

Patients who used PIAP between 1 and 5 years present an OR of 1.16, CI 95% 1.01–1.33 while those who used PIAP for a period longer than 5 years have an OR of 1.5, CI 95% 1.33–1.7. Having between 500 and 999 DDDs presents a risk of 1.01, CI 95% 0.86–1.2, between 1000 and 1999 DDDs 1.34, CI 95% 1.16–1.56 and people who have more than 2000 DDDs had a risk of 1.14, 1.24–1.6.

#### 3.3.3. Prolactin-Sparing Antipsychotic

PSAPs are unclearly associated with BC. Having used PSAPs between 1 and 5 years reports a risk associated OR as 1.17, CI 95% 0.98–1.4 while using them for more than 5 years report a risk of 1.07, CI 95% 0.89–1.28. Possessing between 500 and 999 DDDs is associated with an OR 1.00, CI 95% 0.34–1.74, between 1000 and 1999 the OR is 1.12, 0.87–1.44 and more than 2000 DDDs 1.03–0.86–1.25.

#### 3.3.4. Histological Subtypes and Demographic Factors

Analysis of histological subtypes revealed a significant divergence in vulnerability to long-term antipsychotic exposure. Ductal breast cancer demonstrated the strongest association with prolonged AP use (exceeding 5 years), with an OR of 1.92 (95% CI: 1.36–2.72). Although the association for lobular breast cancer remained statistically significant after five years, the effect size was notably lower (OR 1.41, 95% CI: 1.22–1.63). In both histotypes, shorter exposure durations (1–5 years) did not reach statistical significance, indicating that the oncogenic stimulus, likely mediated by sustained hyperprolactinemia, requires a prolonged latency period to result in clinically detectable invasive disease.

When stratified by demographic factors, the risk profile demonstrated a remarkable consistency across the age spectrum, contradicting the hypothesis that age-related hormonal shifts might mitigate the impact of APs. For patients treated for more than 5 years, the risk remained significantly elevated across all cohorts: those aged < 55 years had an OR of 1.58, those aged 55–69 an OR of 1.42, and those aged > 70 an OR of 1.55.

This trans-generational risk suggests that mammary tissue sensitivity to drug-induced endocrine disruption persists into older age. While younger patients may be more susceptible to prolactin’s mitogenic effects due to higher baseline glandular activity, the continued risk in elderly populations highlights the need for vigilant oncological surveillance regardless of menopausal status or chronological age.

## 4. Heterogeneity and Publication Bias

To evaluate the statistical consistency of our findings, we utilized Galbraith plots to visually inspect inter-study heterogeneity and identify potential outliers that might disproportionately influence the pooled estimates. This approach allowed for a systematic exploration of variance sources beyond what is captured by standard I2 statistics.

Additionally, the potential for publication bias was rigorously assessed using Funnel plots, which illustrate the relationship between effect sizes and their standard errors. Funnel asymmetry was evaluated as an indicator of missing small-study effects or reporting biases. By combining graphical assessments with study-quality audits, this dual-layered approach ensures that the meta-analytic conclusions are based on a precise and transparent evaluation of the available observational evidence, adhering to the highest standards of evidence-based synthesis. Plots are not showed due to high similarity.

## 5. Discussion

The management of psychiatric disorders has become a complex clinical challenge [25,26]. The focus has shifted from solely addressing acute symptomatology to balancing psychotropic efficacy with long-term metabolic and oncological safety. In this context, clinicians often operate within a “grey zone,” where evidence-based decisions must be weighed against the emerging risks of chronic pharmacological exposure. This meta-analysis addresses a critical knowledge gap at the intersection of clinical psychiatry and preventive oncology. The investigation of the association between antipsychotics (APs) and breast cancer (BC) extends beyond statistical correlation and challenges current standards of care regarding the long-term safety profiles of treatments administered to millions of women worldwide.

The non-linear escalation of risk observed after the five-year mark strongly supports the “promotion” hypothesis in mammary carcinogenesis. Unlike direct mutagens, which initiate DNA damage rapidly, hormonal promoters such as prolactin (PRL) act by accelerating the proliferation of preneoplastic clones [27,28]. The prolonged D2 receptor antagonism inherent in chronic AP therapy leads to sustained disinhibition of lactotroph cells in the anterior pituitary [29]. This chronic hyperprolactinemia does not merely alter the systemic hormonal milieu; it fundamentally rewires the local microenvironment of the breast tissue.

Our finding of a nearly doubled risk for ductal carcinoma (OR 1.92) compared to the more modest elevation in lobular carcinoma (OR 1.41) is particularly salient, given that ductal lesions represent the vast majority of invasive breast cancers diagnosed clinically.

From a biological standpoint, this suggests a differential sensitivity of ductal epithelial cells to PRL-mediated signalling. Prolactin receptors are known to activate the JAK2/STAT5 and MAPK/ERK pathways, which collectively drive cell cycle progression and inhibit pro-apoptotic signals [30,31,32].

It is plausible that ductal cells, which are the primary site for the majority of breast malignancies, possess a lower threshold for hormonal saturation, beyond which homeostatic repair mechanisms are overwhelmed as a “biological fatigue.”

Furthermore, the robust dose–response relationship identified in our cohort, climbing from an OR of 1.23 for lower dosages to an OR of 1.44 for heavy exposure (>2000 DDDs), fulfils the essential “Hill’s Criteria” for biological causality [33]. This direct correlation between cumulative pharmacological load and disease probability suggests that the stimulus’s intensity is as critical as its duration. The convergence of time-dependent risk and dose-dependent escalation supports the hypothesis that iatrogenic hyperprolactinemia acts as a relentless promoter of mammary carcinogenesis, particularly when cumulative exposure exceeds the physiological resilience of the ductal epithelium.

The sharp divergence between prolactin-increasing and prolactin-sparing agents represents the interpretative cornerstone of this study. Our results demonstrate that BC risk is not a class-wide property of antipsychotics but is specifically tethered to the drug’s affinity for D2 receptors in the tuberoinfundibular pathway [21].

PIAPs induce a sustained disruption of the endocrine axis, converting what is often dismissed as a transient side effect into a chronic, systemic biochemical signal.

At the molecular level, prolactin functions as a potent mitogen and survival factor for mammary epithelial cells. Upon binding to its receptor, it triggers the JAK2/STAT5 and MAPK/ERK cascades, downstream signalling pathways that are well-documented drivers of cell cycle progression and potent inhibitors of programmed cell death [19,20,22,23] (apoptosis). The data illustrate a clear biological divide: while PIAPs demonstrated a dose-dependent escalation in risk, PSAPs maintained a neutral safety profile even in long-term cohorts (>5 years). This neutrality is a critical finding, as it addresses the issue of confounding by indication. The absence of increased risk in the PSAP group suggests that psychiatric illness itself, or shared lifestyle factors such as obesity and sedentary behaviour, are not the primary drivers of the observed oncological risk. Instead, the evidence implicates the iatrogenic hormonal environment created by specific dopaminergic blockade. This distinction shifts the clinical choice of antipsychotics from a purely symptomatic decision to a pivotal act of preventive neuropsychopharmacology.

This meta-analysis has few limitations: the evidence base is almost entirely observational, residual confounding for key breast cancer risk factors remains substantial, and exposure, outcome, and latency definitions are heterogeneous across studies, limiting causal inference. In addition, the PIAP/PSAP dichotomy, although ATC-based and biologically motivated, simplifies intra-class variability and does not allow precise drug-specific estimates. Strengths include the very large sample size, the explicit integration of duration and cumulative DDDs, the mechanistically informed separation of prolactin-increasing versus prolactin-sparing agents, and the use of PRISMA, MOOSE, mNOS and GRADE frameworks, which together provide a transparent and clinically oriented synthesis of the current observational evidence.

## 6. Conclusions and Clinical Recommendations

This meta-analysis suggests that prolonged antipsychotic use, particularly beyond five years, may be associated with a modest but potentially clinically relevant increase in breast cancer risk, especially for prolactin-increasing agents. Given the predominantly observational nature of the available studies and the low overall certainty of evidence, these findings should be interpreted with caution and viewed as hypothesis-generating rather than definitive. Nevertheless, the consistent signal for higher risk at longer durations and higher cumulative doses together with the stronger association observed for ductal carcinoma, supports a prudent, individualized approach in psychiatric practice, in which the potential oncological implications of long-term prolactin-elevating therapy are discussed and weighed against therapeutic benefits.

## Figures and Tables

**Table 1:** Characteristics of included studies and availability of data on patients with BC receiving Aps *.

Author, Year	Country	Study Design	Mean Age/Age Range (Yrs)	Sample Size	Study Period	Inclusion/Exclusion Criteria	Methods of Validated Unified Exposition	Data Source/Study Database	Adjustment for Covariates
**Chou et al., 2017 [16]**	Taiwan	Population-based retrospective cohort study	Schizophrenia cohort: mean 41.4 (SD 14.3); Non-schizophrenia cohort: mean 42.0 (SD 15.3)	Total N = 40,368 (29,641 schizophrenia; 59,282 controls matched 1:2 → actual analyzed N = 10,727 schizophrenia + 10,727 controls after exclusions	1998–2008 follow-up through 2011	Females with newly diagnosed schizophrenia (ICD-9-CM 295) and prescribed antipsychotic medications (1998–2008). Controls: women without mental illness (ICD-9-CM 290–319) and no antipsychotic drug use. Exclusion: breast cancer (ICD-9-CM 174) before index date or within 1 year follow-up.	Exposure: Schizophrenia diagnosis (ICD-9-CM 295) and antipsychotic prescription validated via National Health Insurance (NHI) claims; categorized by drug type (FGA only, SGA only, FGA + SGA combination) and dose (tertile-based annual mean exposure in g/year). Outcome: Breast cancer (ICD-9-CM 174) identified via linkage to Registry for Catastrophic Illness Patient Database (RCIPD), requiring validated catastrophic illness certification. Propensity score matching used to balance baseline covariates.	Taiwan National Health Insurance Research Database (NHIRD): Longitudinal Health Insurance Database 2000 (LHID2000) and Registry for Catastrophic Illness Patient Database (RCIPD), 1997–2011	Multivariable Cox proportional hazards models adjusted for: age, occupation (white/blue collar, others), monthly income (NTD categories), comorbidities (hypertension, hyperlipidemia, diabetes), and medications (lithium, valproate sodium, antidepressants, anxiolytics/hypnotics). Propensity score matching at enrollment controlled for same baseline variables.
**George et al., 2020 [17]**	USA	Prospective cohort study	Postmenopausal women aged 50–79 at enrollment; mean baseline age about 63 years (psychotropic users ≈ 62, non-users ≈ 63)	155,737 postmenopausal women (analytic cohort after excluding women with prior breast cancer and those with <1 day of follow-up from 161,808 initially enrolled)	Enrollment 1993–1997; follow-up through 31 March 2018 (mean follow-up 14.8 years)	Postmenopausal women aged 50–79 years enrolled in WHI OS or CT; no personal history of breast cancer at baseline; at least 1 day of follow-up	Exposure: psychotropic medication use (any, typical antipsychotics, atypical antipsychotics, lithium) assessed at baseline by direct inventory of all prescription and non-prescription medications brought to the visit; typical vs. atypical antipsychotics classified based on pharmacologic characteristics. Outcome: incident invasive and in situ breast cancer centrally adjudicated using medical records.	Women’s Health Initiative cohort (observational study and clinical trial arms), with centrally adjudicated breast cancer outcomes and detailed baseline medication inventory.	Cox proportional hazards models adjusted for age, WHI component (observational vs. clinical trial), and hormone therapy trial arm; additional baseline variables were evaluated but not retained because they did not materially change hazard ratio estimates.
**Hippisley-Cox et al., 2007 [18]**	United Kingdom	Population-based nested case–control study within an open cohort	Adults 25–100 years; median age varies by cancer type (e.g., breast ≈ 61 years, colon ≈ 72 years, respiratory ≈ 71 years)	Underlying cohort: 4,040,494 patients (18,772,868 person-years). Overall: 40,441 incident cancer cases (including 10,535 breast cancer cases) and up to 5 matched controls per case (e.g., 50,074 controls for breast cancer).	1 January 1995–1 July 2005 (10-year study window using QRESEARCH version 7 with data up to 1 August 2005)	Patients aged 25–100 years registered ≥12 months with participating practices; incident first-ever diagnosis of one of six cancers (breast, colon, rectal, gastroesophageal, prostate, respiratory) during the study period. Controls: cancer-free at index date, matched by single year of age, sex, practice, and calendar time, alive and registered at the matched case’s index date. Exclusions: any prior cancer before index date; for breast cancer, prior mastectomy or tamoxifen >12 months before diagnosis; analogous exclusions for controls with such histories.	Exposure: schizophrenia and bipolar disorder identified from recorded diagnoses ≥12 months before index date; medication exposure based on ≥1 prescription before index date (excluding the 12 months immediately preceding) for antipsychotics (conventional, atypical, lithium) and other relevant drugs (NSAIDs, COX-2 inhibitors, aspirin, statins, hormone therapy, oral contraceptives, SSRIs, TCAs). Outcome: incident cancers identified from coded diagnoses in primary care electronic records; incident status supported by exclusion of prior cancer or prior mastectomy/tamoxifen for breast cancer. Additional standardized variables: latest recorded BMI, smoking status, and Townsend score for deprivation prior to index date.	QRESEARCH general practice database (version 7), comprising anonymized electronic medical records from 454 UK general practices using the EMIS clinical system.	Conditional logistic regression models (matched on age, sex, practice, calendar time) adjusted for smoking status, BMI, socioeconomic status (Townsend score), comorbidities (ischemic heart disease, diabetes, hypertension, rheumatoid arthritis), and prescribed medications (NSAIDs, COX-2 inhibitors, aspirin, statins, SSRIs, TCAs, antipsychotics). Breast, colon, and rectal cancer models additionally adjusted for hormone therapy and oral contraceptive use; models also adjusted for the other mental health condition (schizophrenia vs. bipolar).
**Pottegård et al., 2018 [19]**	Denmark	Nationwide population-based case–control study	Women aged 18–85 years at index date; median age 62 years (IQR 53–70) among breast cancer cases and matched controls	60,360 histologically verified incident invasive breast cancer cases diagnosed 2000–2015; 603,600 female population controls (10 per case) frequency-matched by birth year	Cancer diagnoses 2000–2015; exposure and covariate history from 1995 onward with at least 10 years of residency in Denmark and ≥5 years of prescription data before index date (1-year exposure lag applied)	Female residents of Denmark with first-time invasive breast cancer diagnosis in 2000–2015, aged 18–85 years, continuously resident in Denmark for the prior 10 years, with no previous cancer (except non-melanoma skin cancer) or mastectomy. Controls: cancer-free women from the general population, born in the same year as the case, eligible at index date, selected by risk-set sampling (10 controls per case) and assigned the same index date.	Exposure: antipsychotic use quantified from the National Prescription Registry, standardized to cumulative olanzapine equivalents from 1995 until 1 year before index date; categorized as ever use, long-term use (≥10,000 mg olanzapine equivalents), and finer cumulative exposure bands (0–4999; 5000–9999; 10,000–19,999; 20,000–49,999; ≥50,000 mg). Antipsychotics further classified as first- vs. second-generation and prolactin-inducing vs. non-inducing based on ATC codes and pharmacologic data. Outcome: first invasive breast cancer identified from the Danish Cancer Registry, restricted to histologically verified cases with morphology subtypes and estrogen receptor status from the Danish Pathology Register. Standardized definitions applied to comorbidities, psychiatric diagnoses, drug exposures, and education using national registry coding schemes and the Charlson comorbidity index.	Danish Cancer Registry; National Prescription Registry; National Patient Register; Danish Pathology Register; Danish Psychiatric Central Register; Statistics Denmark registries on education and income; Danish Civil Registration System.	Conditional logistic regression (matched by age and calendar time via risk-set sampling) adjusted for: use of multiple co-medications affecting breast cancer risk (low-dose aspirin, non-aspirin NSAIDs, digoxin, statins, spironolactone, oral steroids, metoclopramide, domperidone, loop diuretics, beta-blockers, vascular calcium-channel blockers, oral contraceptives, hormone replacement therapy—with cumulative use and recency—SSRIs); comorbidities (diabetes, COPD, alcohol-related disease); psychiatric diagnoses (schizophrenia, other psychoses, bipolar and other mood and anxiety/stress-related disorders); Charlson comorbidity index (0, 1–2, ≥3); and highest achieved education as a socioeconomic indicator. A 1-year lag was applied to all exposure and confounder definitions.
**Rahman et al., 2022 [20]**	United States	Large retrospective observational cohort study	Women aged 18–64 years; median age at index ≈ 41 years in antipsychotic group and 39 years in anticonvulsant/lithium group; median age at breast cancer diagnosis ≈ 53 years	540,737 women who were new users of antipsychotics, anticonvulsants, and/or lithium: 914 incident invasive breast cancer cases (0.16% of cohort) during follow-up	Commercial database: 1 January 2007–30 June 2016; Medicaid database: 1 January 2012–30 June 2016; maximum follow-up truncated at 6 years after index date, with ≥12 months pre-index enrollment required	Women 18–64 years with at least one outpatient prescription claim (days’ supply > 0) for an antipsychotic, anticonvulsant, or lithium within the study period; continuous medical and pharmacy enrollment for ≥12 months before first study drug (new-user definition); no evidence before index of breast cancer, history of breast cancer, or tamoxifen use; at least some follow-up after index (until outcome or censoring).	Exposure: antipsychotics classified into three categories by prolactin-elevating potential (category 1 high, category 2 intermediate, category 3 low/none) based on clinical pharmacology literature; comparator drugs were anticonvulsants and lithium (non-prolactin-elevating). Drug exposure quantified using WHO-defined daily doses (DDD) calculated from strength, quantity, and days’ supply on each claim; cumulative DDD from index until outcome/censoring (up to 6 years) divided by observed days to obtain average daily DDD per category. Women were required to be “new users” (no study drug in prior 12 months). Outcome: incident invasive breast cancer identified by hierarchical algorithm using ICD-9/10 diagnosis codes plus evidence of pathology (surgical pathology CPT codes) and/or surgical or chemotherapy treatment codes to confirm invasive disease; likely prevalent or unverified cases (diagnosis without supporting treatment) treated as censoring.	IBM MarketScan Commercial Claims and Encounters Database and IBM MarketScan Multi-State Medicaid Database (U.S. administrative claims with medical and outpatient pharmacy data)	Cox proportional hazards models estimating hazard ratios per 1-unit increase in average daily DDD, adjusted in stages: (1) age only (cubic spline); (2) known breast cancer risk factors (obesity and obesity proxies, diabetes, alcohol abuse, benign breast disease, hormone replacement therapy by type, smoking and smoking-related diagnoses, use of antidiabetic, lipid-lowering and smoking-cessation drugs, plus Medicaid enrollment as proxy for parity/age at first birth); (3) age plus these risk factors; and sensitivity models additionally adjusted for psychiatric diagnoses (bipolar disorder, schizophrenia, major depression). Separate models run for pooled antipsychotics and for categories 1–3, with stratified analyses by age (≤50 vs. 51–64 years).
**Solmi et al., 2024 [21]**	Sweden	Nested case–control study within a nationwide cohort of women	Women aged 18–85 years at breast cancer diagnosis; mean age at diagnosis ≈ 63.3 years (SD~11.8)	Source cohort: 132,061 women with schizophrenia, schizoaffective disorder, other non-affective psychotic disorders, or bipolar disorder. Cases: 1642 incident breast cancer cases (1.24%) between 2010 and 2021. Controls: 8173 matched controls (up to 5 per case).	Psychiatric diagnoses identified 2006–2021; exposure and covariates from 2005 onward (start of Prescribed Drug Register); breast cancer inclusion period 1 January 2010–31 December 2021, ensuring ≥4.5 years of exposure history before diagnosis; 1-year lag for exposure.	Women with at least one diagnosis of schizophrenia/schizoaffective disorder, other non-affective psychotic disorder, or bipolar disorder; first incident malignant breast cancer (ICD codes) between 2010 and 2021; age 18–85 at diagnosis; ≥4.5 years of antipsychotic exposure data before index; no prior cancer (except non-melanoma skin), HIV, mastectomy, or organ transplant. Controls: women from same psychiatric cohort, cancer-free at index, matched to cases by age (±1 year), primary psychiatric diagnosis, and time since first psychiatric diagnosis (±1 year), with same exclusion criteria.	Exposure: antipsychotic drugs (ATC N05A, excluding lithium) classified as prolactin-increasing vs. prolactin-sparing based on prior pharmacologic evidence. Drug utilization from the Prescribed Drug Register (dispensing date, ATC, strength, package size, DDDs) converted to continuous exposure periods using the PRE2DUP method, accounting for stockpiling and hospital stays. Prolactin-sparing exposure defined only when used without concomitant prolactin-increasing antipsychotics; otherwise counted as prolactin-increasing. Cumulative exposure categorized by duration (<1 year, 1–<5 years, ≥5 years) and by cumulative DDD bands (<500, 500–<1000, 1000–<2000, ≥2000 DDD). A 1-year lag window before index-excluded recent exposure to limit reverse causation. Outcome: incident malignant breast cancer from the Swedish Cancer Register with morphology (ductal, lobular, other) and diagnosis date; first diagnosis only.	Swedish National Patient Register (inpatient and specialized outpatient care); MiDAS register (sickness absence and disability pension); Prescribed Drug Register (outpatient dispensings); Swedish Cancer Register (breast cancer diagnoses and histology); population and social registers (for linkage and number of children).	Conditional logistic regression (matched sets as strata), adjusted for somatic comorbidities (cardiovascular disease, asthma/COPD, diabetes), psychiatric comorbidities (substance use disorder, prior suicide attempt), reproductive proxy (number of children), systemic hormone replacement therapy (estrogen/progestogen use and cumulative duration), and co-medications potentially related to breast cancer risk (angiotensin-system drugs, beta-blockers, dihydropyridine calcium-channel blockers, digitalis, loop diuretics, spironolactone, statins, opioids, paracetamol, anticholinergic antiparkinsonian drugs, SSRIs, TCAs, metformin). Models for prolactin-increasing and prolactin-sparing antipsychotics mutually adjusted for each other; additional sensitivity analyses stratified by cancer type, psychiatric diagnosis subgroup, age group, and excluding aripiprazole time from prolactin-increasing exposure.
**Taipale et al., 2021 [22]**	Finland	Nationwide nested case–control study within a cohort of women	Women aged 18–85 years at breast cancer diagnosis; mean age ≈ 62.2 years (SD~10.3) for both cases and matched controls	Source cohort: 30,785 women with schizophrenia (≥16 years). Cases: 1069 incident invasive breast cancer cases (3.5%) diagnosed 2000–2017. Controls: 5339 women with schizophrenia without breast cancer (up to 5 per case), matched by age and illness duration	Schizophrenia diagnoses 1972–2014; prescription data from 1995 onward; breast cancers included from 1 January 2000 to 31 December 2017, ensuring ≥5 years of exposure history; 1-year lag for exposure and covariates (sensitivity analyses with 0- and 3-year lags)	Women with schizophrenia identified in the Hospital Discharge Register (ICD-8/9 295, ICD-10 F20/F25); first histologically verified invasive breast cancer after schizophrenia diagnosis between 2000 and 2017; age 18–85 at diagnosis; ≥5 years of prescription follow-up before index. Exclusions (before index): any prior cancer (except non-melanoma skin), organ transplant, mastectomy, or HIV. Controls: women with schizophrenia from same base cohort, cancer-free at index, matched 1:5 on age (±1 year) and time since first schizophrenia diagnosis (±1 year), with same exclusions; controls could later become cases	Exposure: all antipsychotics (ATC N05A, excluding lithium) grouped as prolactin-increasing vs. prolactin-sparing (clozapine, quetiapine, aripiprazole) based on prior pharmacologic evidence. Periods of use were derived from the national prescription register using the PRE2DUP algorithm, which converts dispensing histories (dates, strengths, package sizes, defined daily doses) into continuous drug-use periods accounting for stockpiling and hospital stays. Cumulative duration of use for each group categorized as <1 year (reference, including never/short use), 1–4 years, and ≥5 years; for some analyses further split (5–9, 10–14, ≥15 years). Cumulative dose summarized as total defined daily doses (<500, 500–999, 1000–1999, 2000–4999, ≥5000) and as average defined daily dose per day, with a 1-year lag window before index to minimize reverse causation. Prolactin-sparing exposure counted only when not overlapping prolactin-increasing use; concurrent use was attributed to the prolactin-increasing category. Sensitivity analyses excluded periods of concomitant aripiprazole from prolactin-increasing exposure. Outcome: first incident invasive breast cancer from the Finnish Cancer Registry, histologically verified, coded by ICD-10 and oncology morphology, allowing classification into ductal and lobular adenocarcinoma and stage (localized, non-localized, unknown).	Finnish Hospital Discharge Register (inpatient and specialist outpatient care); national Prescription Register (reimbursed outpatient prescriptions, with ATC and defined daily doses); Finnish Cancer Registry (all cancers since 1953 with morphology and stage); all linked via unique personal identification numbers.	Conditional logistic regression stratified by matched sets, adjusted for: somatic comorbidities (cardiovascular disease, diabetes, asthma/chronic obstructive pulmonary disease), psychiatric history (substance misuse, prior suicide attempt), reproductive proxy (number of children), systemic hormone replacement therapy (estrogen/gestagen preparations, with cumulative duration categorized as non-use, <1 year, 1–4 years, ≥5 years), and multiple co-medications that may influence breast cancer risk (beta-blockers, dihydropyridine calcium-channel blockers, angiotensin-system drugs, digoxin, spironolactone, loop diuretics, statins, non-steroidal anti-inflammatory drugs, opioids, paracetamol, anticholinergic antiparkinsonian drugs, tricyclic antidepressants, selective serotonin reuptake inhibitors, verapamil). Models for prolactin-increasing and prolactin-sparing antipsychotic exposures mutually adjusted for each other; further sensitivity analyses- varied lag windows and exposure parameterization (duration, cumulative defined daily doses, dose).
**Chu et al., 2023 [23]**	Hong Kong SAR, China	Nested case–control study	Bipolar disorder cases: mean 52.94 years (SD 11.55); bipolar controls: 52.18 years (SD 10.87). Schizophrenia cases: mean 57.92 years (SD 11.68); schizophrenia controls: 57.94 years (SD 11.60).	Total: 672 cases (109 with bipolar disorder, 563 with schizophrenia) and 6450 controls (931 with bipolar disorder, 5519 with schizophrenia). Underlying cohort: 14,913 women with bipolar disorder and 68,708 women with schizophrenia first diagnosed 1999–2018.	Underlying cohort: women first diagnosed with schizophrenia or bipolar disorder between 1 January 1999, and 31 December 2018; followed until first breast cancer diagnosis, death, or end of data availability. Electronic health records available since 1993, transferred daily to database.	Women aged ≥18 years first diagnosed with schizophrenia (ICD-9-CM 295.0–295.9) or bipolar disorder (ICD-9-CM 296.1, 296.4–296.8) between 1999 and 2018 by licenced psychiatrists in Hong Kong public healthcare facilities. Exclusion: missing date of birth or sex, history of breast cancer, diagnosed with both schizophrenia and bipolar disorder (to avoid misclassification), aged <18 at index date. Cases: women in underlying cohort with first breast cancer diagnosis (ICD-9-CM 174.0–174.9). Controls: up to 10 matched per case by birth year and healthcare setting (inpatient/outpatient), without breast cancer and alive, using incidence density sampling with replacement. Index date: breast cancer diagnosis date for matched sets.	Exposure: antipsychotic use categorized as first-generation antipsychotics (FGA) and second-generation antipsychotics (SGA) based on dispensing records. Exposure defined as >1 year of FGA or SGA use versus non-use (none or <1 year). Generic names used to identify medications. Sensitivity analysis: duration further categorized as non-use, 1–4 years, ≥5 years. Outcome: breast cancer diagnosis (ICD-9-CM 174.0–174.9); secondary outcomes examined specific tumour locations. Exposure and covariates ascertained from records before index date.	Hospital Authority Clinical Data Analysis and Reporting System: territory-wide electronic health record system covering all Hong Kong public hospitals (sole provider of public inpatient services, major outpatient provider). Database includes demographics, clinical diagnoses, medication dispensing records, diagnosis settings since 1993. Public healthcare covers >83% of Hong Kong residential population at negligible out-of-pocket cost. Predominantly ethnic Chinese population.	Multivariable conditional logistic regression adjusted for: time since first psychiatric diagnosis (continuous), clinical history (circulatory diseases excluding hypertension, hypertension, obesity, diabetes, suicide/self-inflicted injury, asthma, alcohol abuse, chronic pulmonary diseases excluding asthma), medication history prior to index date (calcium-channel blocker, loop diuretics, statin, opioid, selective serotonin reuptake inhibitor, serotonin modulators, serotonin–norepinephrine reuptake inhibitor, tricyclic antidepressant, tetracyclic antidepressants, hypnotics, anxiolytic, benzodiazepines, non-steroidal anti-inflammatory drugs). Matching by birth year and healthcare setting. Sensitivity analyses: excluded specific breast cancer subtypes, examined dose–response by duration, excluded participants with history of any other cancer.

* The use of dual colors and bold format enhances the readability of the table.

## Data Availability

The original contributions presented in this study are included in the article/Supplementary Materials. Further inquiries can be directed to the corresponding authors.

## Supplementary Materials

The following supporting information can be downloaded at https://www.mdpi.com/article/10.3390/medsci14020205/s1.

## Abbreviations

AP (antipsychotic), BC (breast cancer), CI (confidence interval), HR (hazard ratio), ICD (International Classification of Diseases), mNOS (modified Newcastle-Ottawa Scale), and OR (odds ratio). Additional methodological and pharmacological abbreviations include DDD (Defined Daily Dose), PIAPs (prolactin-increasing antipsychotics), PSAPs (prolactin-sparing antipsychotics), and ATC (Anatomical Therapeutic Chemical classification system). The reporting and evidence-grading framework introduces PRISMA (Preferred Reporting Items for Systematic Reviews and Meta-Analyses), MOOSE (Meta-analyses of Observational Studies in Epidemiology), RCT(s) (randomized controlled trial(s)), RR(s) (relative risk(s)), PROSPERO (International Prospective Register of Systematic Reviews), and REML (restricted maximum likelihood).
