# Supplementary material for: Breast Cancer Risk in over 1.3 Million Women on Antipsychotic Therapy: Life-Saving Drugs or Hidden Trigger for Breast Cancer?
Source: Med Sci (Basel). 2026 Apr 20;14(2):205. doi: 10.3390/medsci14020205 (PMC13204435; doi:10.3390/medsci14020205)
Supplement: Supplementary file 1 [file medsci-14-00205-s001.zip › medsci-4228601-supplementary.pdf]

SUPPLEMENTARY

Research strategy

PUBMED

(("Neoplasms"[MeSH] OR neoplasm* OR tumor* OR carcinoma*) AND ("Antipsychotic Agents"[MeSH] OR "Antipsychotic Agent"[Title/Abstract] OR "Antipsychotic Agents"[MeSH] OR "Antipsychotic Agent"[Title/Abstract] OR "Tranquilizing Agents"[MeSH])) AND humans[Filter] AND English [Filter]

EMBASE

(('neoplasm'/exp OR 'neoplasm' OR neoplasm:ti,ab,kw OR 'neoplasm'/de) AND 'antipsychotic agent':ti,ab,kw OR 'antipsychotic agent'/de OR 'neuroleptic agent':ti,ab,kw OR 'neuroleptic agent'/de OR 'tranquilizer':ti,ab,kw) AND 'tranquilizer'/de AND english:la AND 'human'/de NOT editorial:it NOT 'conference abstract':it NOT letter:it

SCOPUS

TITLE-ABS-KEY (cancer* OR neoplasm* OR malignant* OR tumour* OR tumor* OR carcinoma* OR adenocarcinoma*) AND TITLE-ABS-KEY (antipsychotic AND agent OR neuroleptic AND agent OR antipsychotic AND agent OR tranquilizing AND agent) AND (LIMIT-TO (EXACTKEYWORD , "Human")) AND (LIMIT-TO (LANGUAGE , "English")) AND (LIMIT-TO (DOCTYPE , "ar") OR LIMIT-TO (DOCTYPE , "re"))

Table S1. PRISMA Checklist

Section and Topic	Item #	Checklist item	Location where item is reported
**TITLE**			
Title	1	Identify the report as a systematic review.	1
**ABSTRACT**			
Abstract	2	See the PRISMA 2020 for Abstracts checklist.	2
**INTRODUCTION**			
Rationale	3	Describe the rationale for the review in the context of existing knowledge.	2-3
Objectives	4	Provide an explicit statement of the objective(s) or question(s) the review addresses.	2-3
**METHODS**			
Eligibility criteria	5	Specify the inclusion and exclusion criteria for the review and how studies were grouped for the syntheses.	3-4
Information sources	6	Specify all databases, registers, websites, organisations, reference lists and other sources searched or consulted to identify studies. Specify the date when each source was last searched or consulted.	3-5
Search strategy	7	Present the full search strategies for all databases, registers and websites, including any filters and limits used.	supplementary
Selection process	8	Specify the methods used to decide whether a study met the inclusion criteria of the review, including how many reviewers screened each record and each report retrieved, whether they worked independently, and if applicable, details of automation tools used in the process.	3-5
Data collection process	9	Specify the methods used to collect data from reports, including how many reviewers collected data from each report, whether they worked independently, any processes for obtaining or confirming data from study investigators, and if applicable, details of automation tools used in the process.	3-5
Data items	10a	List and define all outcomes for which data were sought. Specify whether all results that were compatible with each outcome domain in each study were sought (e.g. for all measures, time points, analyses), and if not, the methods used to decide which results to collect.	3-5
	10b	List and define all other variables for which data were sought (e.g. participant and intervention characteristics, funding sources). Describe any assumptions made about any missing or unclear information.	3-5
Study risk of bias assessment	11	Specify the methods used to assess risk of bias in the included studies, including details of the tool(s) used, how many reviewers assessed each study and whether they worked independently, and if applicable, details of automation tools used in the process.	3-5 supplementary
Effect measures	12	Specify for each outcome the effect measure(s) (e.g. risk ratio, mean difference) used in the synthesis or presentation of results.	3-5
Synthesis methods	13a	Describe the processes used to decide which studies were eligible for each synthesis (e.g. tabulating the study intervention characteristics and comparing against the planned groups for each synthesis (item #5)).	3-5
	13b	Describe any methods required to prepare the data for presentation or synthesis, such as handling of missing summary statistics, or data conversions.	3-5
	13c	Describe any methods used to tabulate or visually display results of individual studies and syntheses.	3-5
	13d	Describe any methods used to synthesize results and provide a rationale for the choice(s). If meta-analysis was performed, describe the model(s), method(s) to identify the presence and extent of statistical heterogeneity, and software package(s) used.	3-5
	13e	Describe any methods used to explore possible causes of heterogeneity among study results (e.g. subgroup analysis, meta-regression).	3-5
	13f	Describe any sensitivity analyses conducted to assess robustness of the synthesized results.	3-5
Reporting bias	14	Describe any methods used to assess risk of bias due to missing results in a synthesis (arising from reporting biases).	3-5-

Section and Topic	Item #	Checklist item	Location where item is reported
assessment			supplementary
Certainty assessment	15	Describe any methods used to assess certainty (or confidence) in the body of evidence for an outcome.	3-5-supplementary
**RESULTS**			
Study selection	16a	Describe the results of the search and selection process, from the number of records identified in the search to the number of studies included in the review, ideally using a flow diagram.	6-10
	16b	Cite studies that might appear to meet the inclusion criteria, but which were excluded, and explain why they were excluded.	6-10
Study characteristics	17	Cite each included study and present its characteristics.	Guideline 1
Risk of bias in studies	18	Present assessments of risk of bias for each included study.	supplementary
Results of individual studies	19	For all outcomes, present, for each study: (a) summary statistics for each group (where appropriate) and (b) an effect estimate and its precision (e.g. confidence/credible interval), ideally using structured tables or plots.	6-10
Results of syntheses	20a	For each synthesis, briefly summarise the characteristics and risk of bias among contributing studies.	6-10
	20b	Present results of all statistical syntheses conducted. If meta-analysis was done, present for each the summary estimate and its precision (e.g. confidence/credible interval) and measures of statistical heterogeneity. If comparing groups, describe the direction of the effect.	6-10-supplementary
	20c	Present results of all investigations of possible causes of heterogeneity among study results.	6-10-supplementary
	20d	Present results of all sensitivity analyses conducted to assess the robustness of the synthesized results.	6-10
Reporting biases	21	Present assessments of risk of bias due to missing results (arising from reporting biases) for each synthesis assessed.	Supplementary
Certainty of evidence	22	Present assessments of certainty (or confidence) in the body of evidence for each outcome assessed.	Supplementary
**DISCUSSION**			
Discussion	23a	Provide a general interpretation of the results in the context of other evidence.	10-11
	23b	Discuss any limitations of the evidence included in the review.	10-11
	23c	Discuss any limitations of the review processes used.	10-11
	23d	Discuss implications of the results for practice, policy, and future research.	10-11
**OTHER INFORMATION**			
Registration and protocol	24a	Provide registration information for the review, including register name and registration number, or state that the review was not registered.	5
	24b	Indicate where the review protocol can be accessed, or state that a protocol was not prepared.	5
	24c	Describe and explain any amendments to information provided at registration or in the protocol.	5
Support	25	Describe sources of financial or non-financial support for the review, and the role of the funders or sponsors in the review.	14

Section and Topic	Item #	Checklist item	Location where item is reported
Competing interests	26	Declare any competing interests of review authors.	2
Availability of data, code and other materials	27	Report which of the following are publicly available and where they can be found: template data collection forms; data extracted from included studies; data used for all analyses; analytic code; any other materials used in the review.	2

Table S2. MOOSE Checklist

Supplementary Table S2. MOOSE (Meta-analyses Of Observational Studies in Epidemiology) Checklist.

Reporting Criteria	Reported (Yes/No)	Reported on Page No.
**Reporting of Background**		
Problem definition	Yes	1
Hypothesis statement	Yes	1-2
Description of Study Outcome(s)	Yes	1-2
Type of exposure or intervention used	Yes	1-2
Type of study design used	Yes	1-2
Study population	Yes	5
**Reporting of Search Strategy**		

Qualifications of searchers (eg, librarians and investigators)	Yes	3-5
Search strategy, including time period included in the synthesis and keywords	Yes	3-5
Effort to include all available studies, including contact with authors	Yes	3-5
Databases and registries searched	Yes	3-5
Search software used, name and version, including special features used (eg, explosion)	Yes	3-5
Use of hand searching (eg, reference lists of obtained articles)	Yes	3-5
List of citations located and those excluded, including justification	Yes	5-suppl
Method for addressing articles published in languages other than English	Yes	3-5
Method of handling abstracts and unpublished studies	Yes	3-5
Description of any contact with authors	Yes	3-5
**Reporting of Methods**		
Description of relevance or appropriateness of studies assembled for assessing the hypothesis to be tested	Yes	3-5
Rationale for the selection and coding of data (eg, sound clinical principles or convenience)	Yes	3-5
Documentation of how data were classified and coded (eg, multiple raters, blinding, and interrater reliability)	Yes	3-5
Assessment of confounding (eg, comparability of cases and controls in studies where appropriate	Yes	3-5- supp
**Reporting Criteria**	**Reported (Yes/No)**	**Reported on Page No.**

Assessment of study quality, including blinding of quality assessors; stratification or regression on possible predictors of study results	Yes	6-7-supp
Assessment of heterogeneity	Yes	6-8
Description of statistical methods (eg, complete description of fixed or random effects models, justification of whether the chosen models account for predictors of study results, dose-response models, or cumulative meta-analysis) in sufficient detail to be replicated	Yes	6-9
Provision of appropriate tables and graphics	Yes	supp
**Reporting of Results**		
Table giving descriptive information for each study included	Yes	6-10
Results of sensitivity testing (eg, subgroup analysis)	Yes	6-10-supp
Indication of statistical uncertainty of findings	Yes	supp
**Reporting of Discussion**		
Quantitative assessment of bias (eg, publication bias)	Yes	supp
Justification for exclusion (eg, exclusion of non−English-language citations)	Yes	21-suppl
Assessment of quality of included studies	Yes	suppl
**Reporting of Conclusions**		
Consideration of alternative explanations for observed results	Yes	10-11
Generalization of the conclusions (ie, appropriate for the data presented and within the domain of the literature review)	Yes	10-11

Guidelines for future research		Yes			10-11	
Disclosure of funding source		Yes			13	

Supplementary Table S3. Quality assessment for studies using the Newcastle–Ottawa Quality Assessment Scale: cohort studies and case-control studies

MODIFIED NEWCASTLE - OTTAWA QUALITY ASSESSMENT SCALE - COHORT STUDIES

Note: A study can be awarded a maximum of one star for each numbered item within the Selection and Outcome categories. A maximum of two stars can be given for Comparability

	Selection				Comparability	Outcome			
**Author, year**	**Representativeness**	**Selection**	**Ascertainment of exposure**	**Demonstration that outcome**	**Variables(s)**	**Assessment of outcome**	**Was follow-up long enough**	**Adequacy of follow-up**	**Overall**
**Chou et al., 2017**	★	★	★	★	★ ★	★	★	★	9
**George et al., 2020**	★	★	★	★		★	★	★	7

Rahman et al., 2022	★	★	★	★	★	★	★	★	8
Wu Chou et al., 2017	★	★	★	★	★	★	★	★	9

MODIFIED NEWCASTLE - OTTAWA QUALITY ASSESSMENT SCALE - CASE-CONTROL STUDIES

Note: A study can be awarded a maximum of one star for each numbered item within the Selection and Exposure categories. A maximum of two stars can be given for Comparability.

		Selection			Comparability		Outcome		
Author, year	Is the case definition adequate?	Representativeness of the cases	Selection of controls	Definition of controls	Comparability of cases and controls on the basis of the design or analysis	Ascertainment of exposure	Same method of ascertainment for cases and controls	Non-response rate	Overall
Chu et al., 2023	★	★	★	★	★	★	★	★	8

**Hippisley-Cox et al., 2007**	★	★	★	★	★	★	★	★	9
**Pottegård et al., 2018**	★	★	★	★	★	★	★	★	9
**Solmi et al., 2024**	★	★	★	★	★	★	★	★	9
**Taipale et al., 2021**	★	★	★	★	★	★	★	★	9

NOTE: For each study, a maximum of one star for each numbered item within the Selection and Exposure categories was awarded. A maximum of two stars was given for Comparability. The total number of stars earned (maximum score of 9) was a measure of study quality.

The Newcastle–Ottawa Scale (NOS) was used to assess the quality of the included studies. For case–control studies, stars are awarded within the Selection domain when the case definition is adequately validated, cases are representative, community controls are used, and controls have no history of the outcome. In the Comparability domain, one star is given if analyses are adjusted for breast cancer and an additional star if further confounders are controlled. In the Exposure domain, stars are assigned for secure ascertainment of exposure (e.g., medical or surgical records), blinded interviews, consistent methods of exposure assessment between cases and controls, and similar response rates. For cohort studies, the

Selection domain awards stars when the exposed cohort is representative of the average population, the nonexposed cohort is drawn from the same community, exposure is ascertained from secure records or structured interviews, and the outcome is absent at baseline. In the Comparability domain, one star is given if analyses adjust for breast cancer and an additional star if further confounders are included. In the Outcome domain, stars are awarded for independent or record-based outcome assessment, sufficient length of follow-up, and adequate follow-up of the cohort. Higher numbers of stars indicate better methodological quality.

Table S4. GRADE Assessment

Grading of Recommendations, Assessment, Development and Evaluation

Author	*Risk of Bias*	*Inconsistency*	*Indirectness*	*Imprecision*	*Publication Bias*	*Overall*
**Chou et al., 2017**	Serious	Not serious	Not serious	Not serious	Undetected	Low
**George et al., 2020**	Serious	Not serious	Not serious	Serious	Undetected	Very low
**Hippisley-Cox et al., 2007**	Not serious	Not serious	Not serious	Not serious	Undetected	Low

**Pottegård et al., 2018**	Not serious	Not serious	Not serious	Not serious	Undetected	Low
**Rahman et al., 2022**	Serious	Not serious	Not serious	Not serious	Undetected	Low
**Solmi et al., 2024**	Not serious	Not serious	Not serious	Not serious	Undetected	Low
**Taipale et al., 2021**	Not serious	Not serious	Not serious	Not serious	Undetected	Low
**Wu Chou et al., 2017**	Not serious	Not serious	Not serious	Not serious	Undetected	Low
**Chu et al., 2023**	Serious	Not serious	Not serious	Serious	Undetected	Very low

CUMULATIVE DURATION

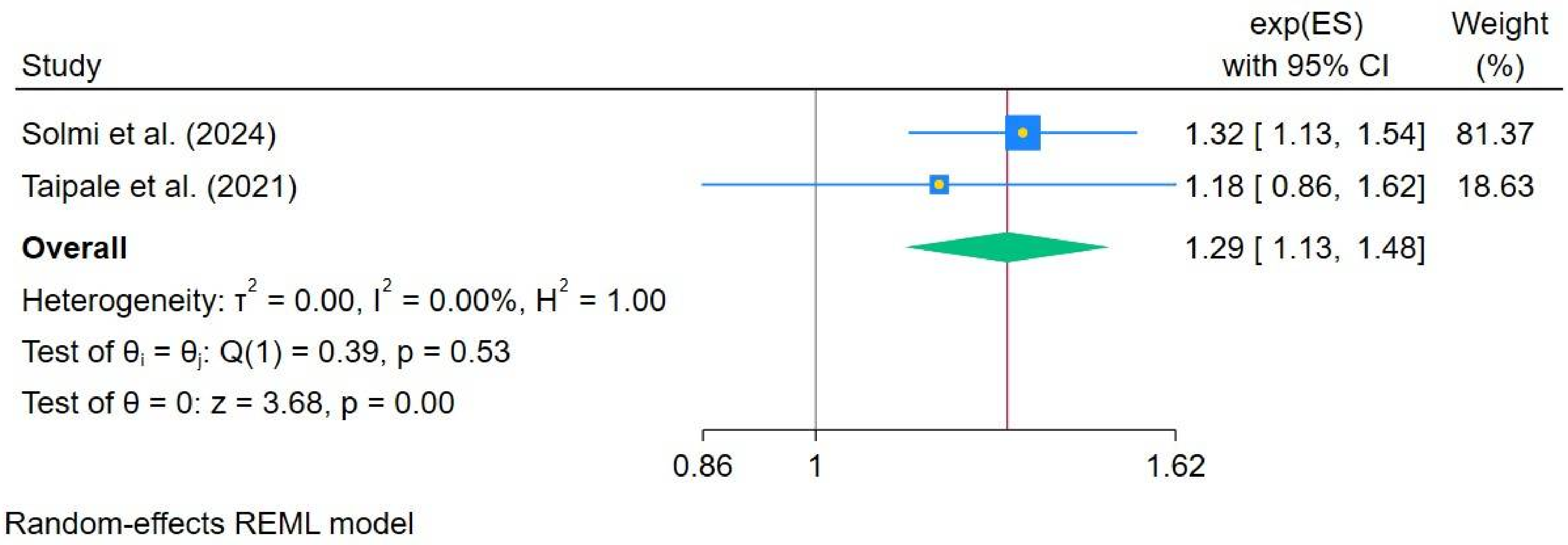

Figure S1. Cumulative Duration - Any APs use 1-5 years

The area of each square is proportional to the sample size of the respective study, with horizontal lines through the squares representing the 95% confidence interval (C.I.) for that study. In the pooled analysis, the diamond symbol represents the pooled estimate, with the right and left points of the diamond indicating the 95% C.I. for the overall analysis

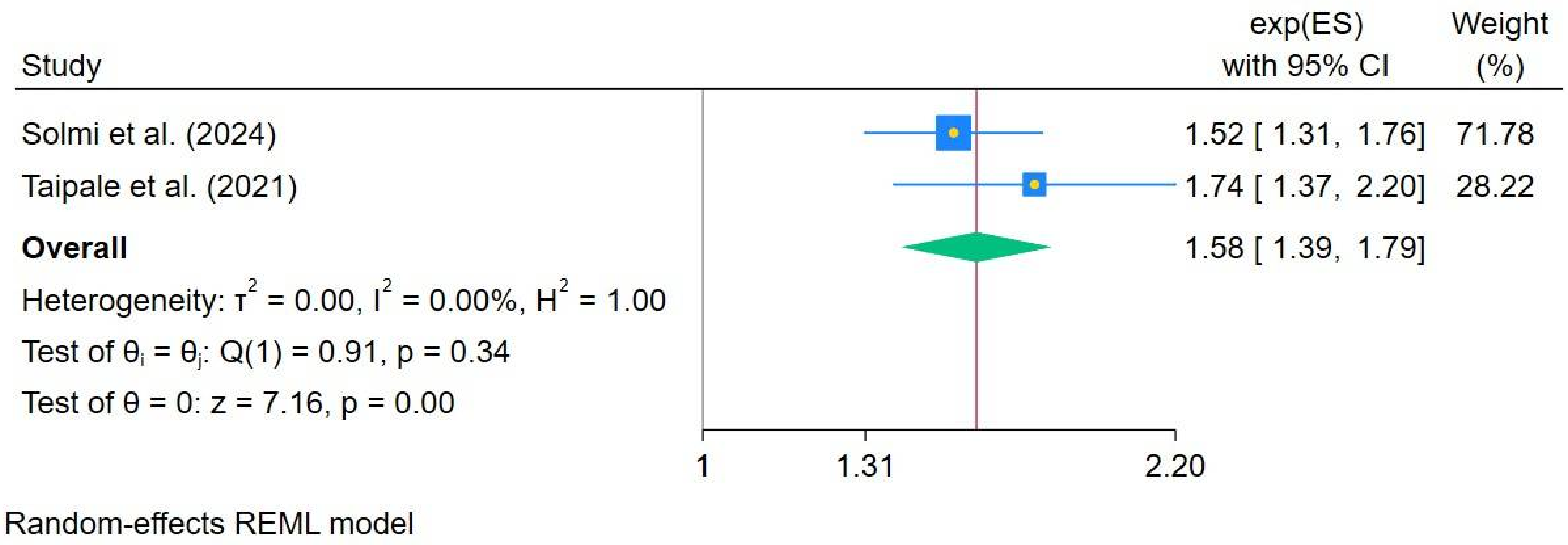

Figure S2. Cumulative Duration - Any APs use >-5 years

The area of each square is proportional to the sample size of the respective study, with horizontal lines through the squares representing the 95% confidence interval (C.I.) for that study. In the pooled analysis, the diamond symbol represents the pooled estimate, with the right and left points of the diamond indicating the 95% C.I. for the overall analysis

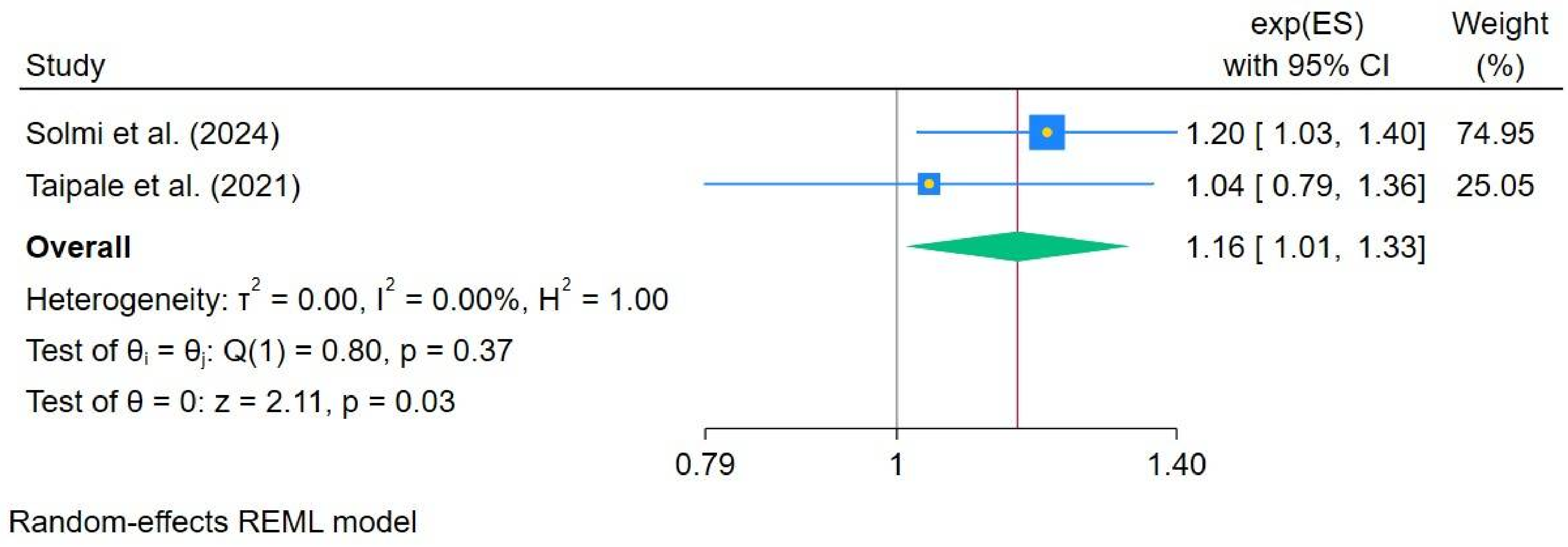

Figure S3. Cumulative Duration – PIAP 1-5 years

The area of each square is proportional to the sample size of the respective study, with horizontal lines through the squares representing the 95% confidence interval (C.I.) for that study. In the pooled analysis, the diamond symbol represents the pooled estimate, with the right and left points of the diamond indicating the 95% C.I. for the overall analysis

PIAP >5 Y

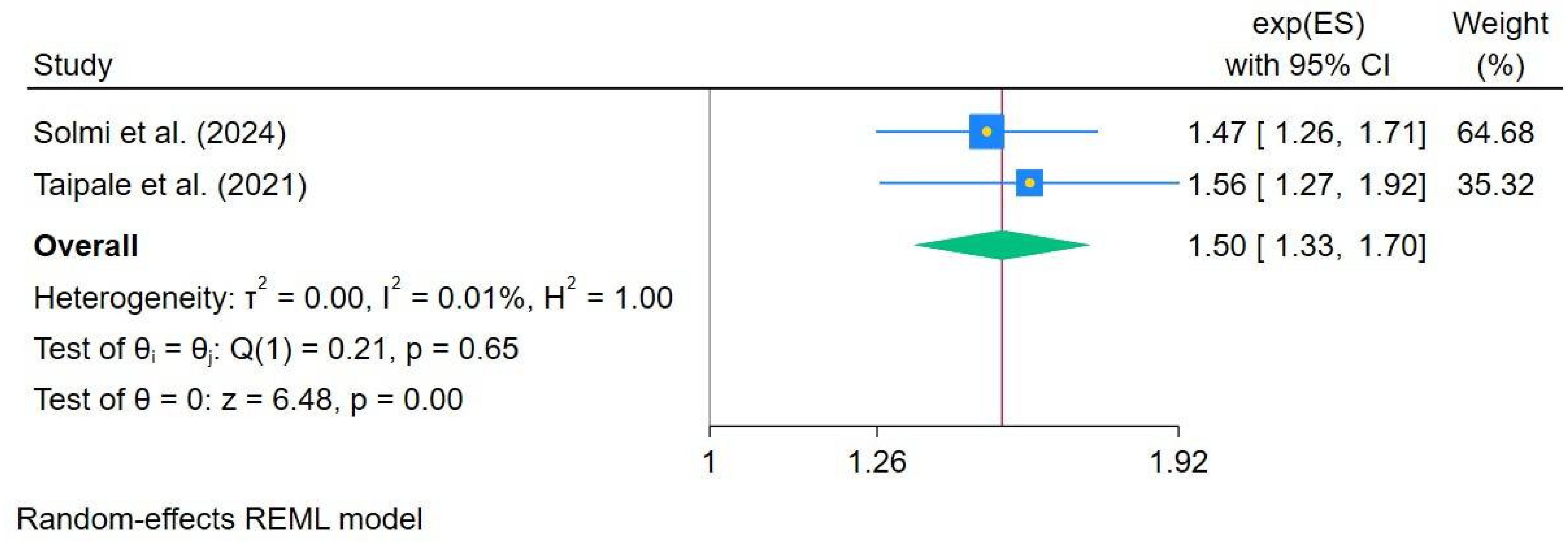

Figure S4. Cumulative Duration – PIAP >5 years

The area of each square is proportional to the sample size of the respective study, with horizontal lines through the squares representing the 95% confidence interval (C.I.) for that study. In the pooled analysis, the diamond symbol represents the pooled estimate, with the right and left points of the diamond indicating the 95% C.I. for the overall analysis

AP SPARING 1 5

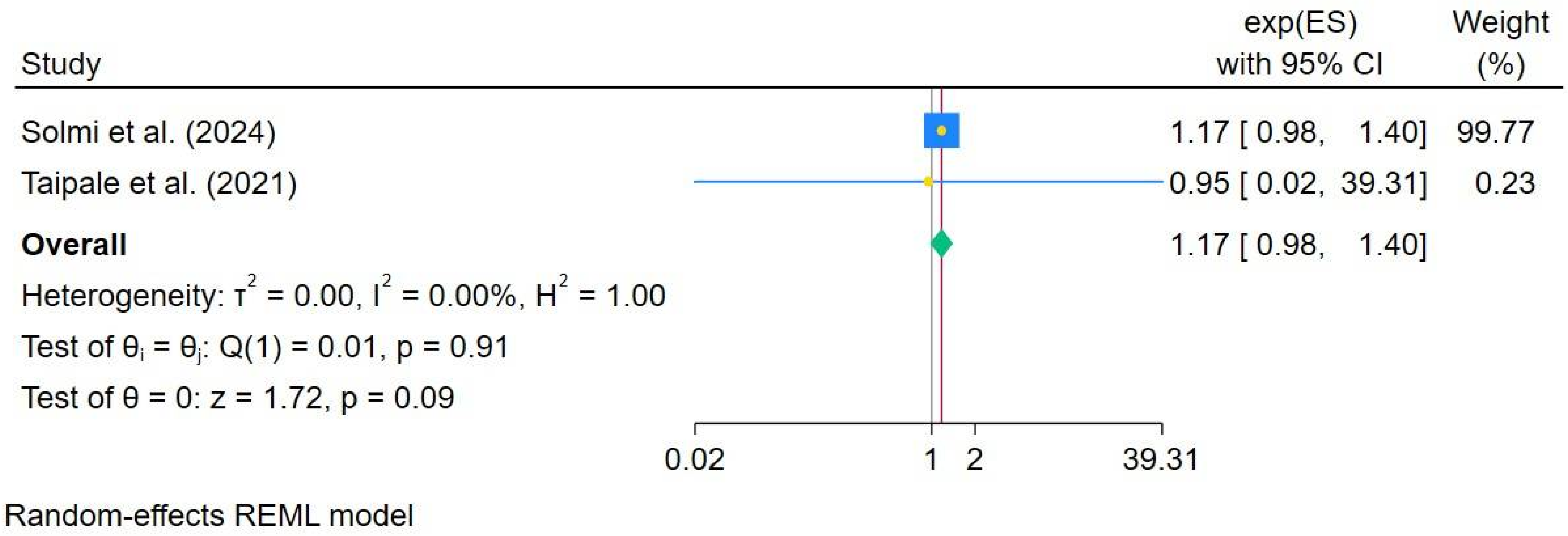

Figure S5. Cumulative Duration – PSAP 1-5 years

The area of each square is proportional to the sample size of the respective study, with horizontal lines through the squares representing the 95% confidence interval (C.I.) for that study. In the pooled analysis, the diamond symbol represents the pooled estimate, with the right and left points of the diamond indicating the 95% C.I. for the overall analysis

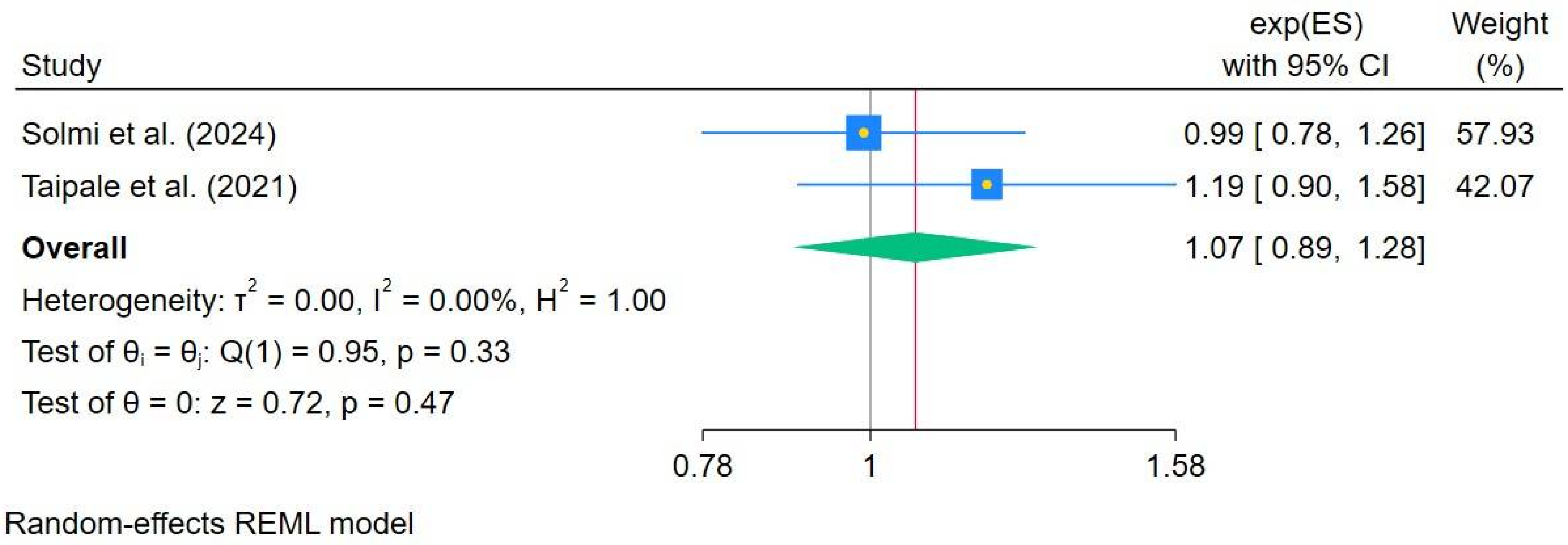

Figure S6. Cumulative Duration – PSAP >5 years

The area of each square is proportional to the sample size of the respective study, with horizontal lines through the squares representing the 95% confidence interval (C.I.) for that study. In the pooled analysis, the diamond symbol represents the pooled estimate, with the right and left points of the diamond indicating the 95% C.I. for the overall analysis

500-999

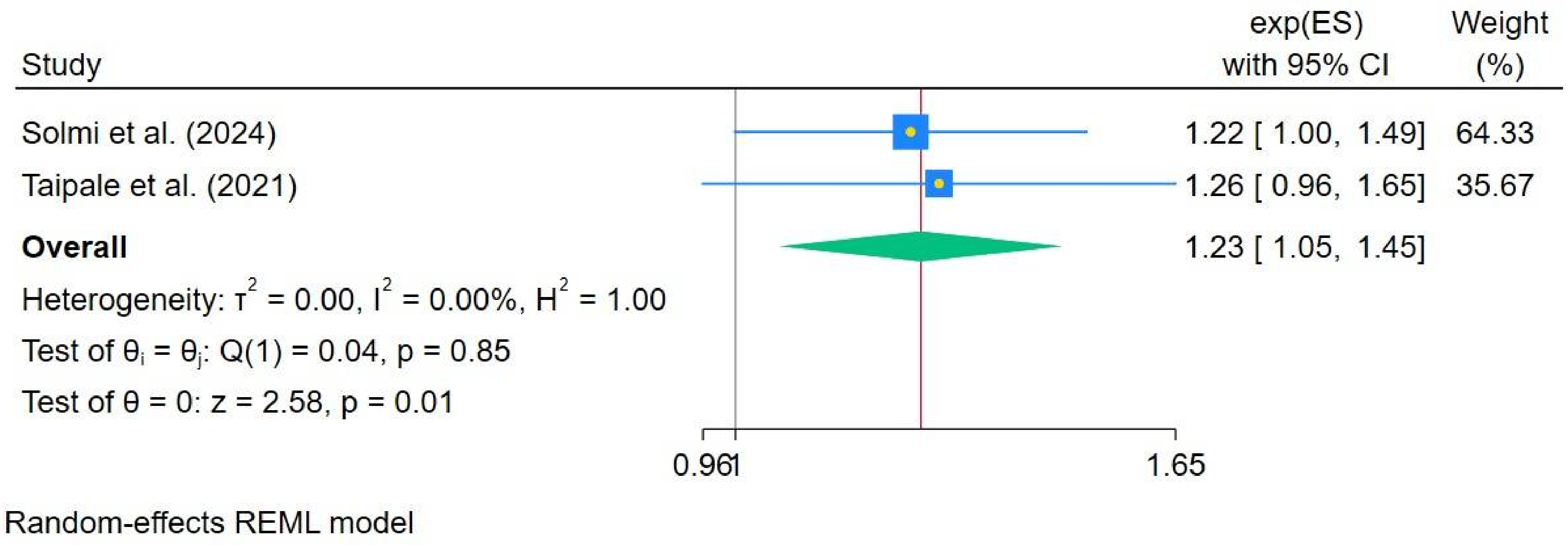

Figure S7. Cumulative Dispensed DDD – Any AP – 500-999 DDD

The area of each square is proportional to the sample size of the respective study, with horizontal lines through the squares representing the 95% confidence interval (C.I.) for that study. In the pooled analysis, the diamond symbol represents the pooled estimate, with the right and left points of the diamond indicating the 95% C.I. for the overall analysis

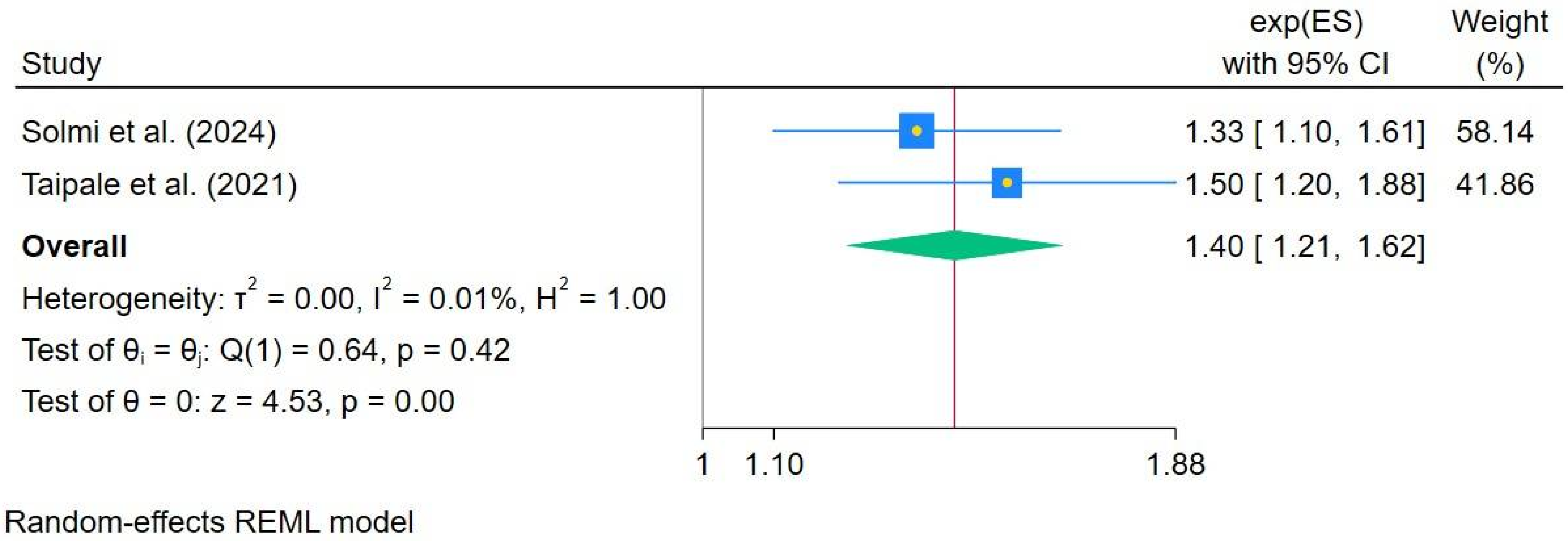

Figure S8. Cumulative Dispensed DDD – Any AP – 1000-1999 DDD

The area of each square is proportional to the sample size of the respective study, with horizontal lines through the squares representing the 95% confidence interval (C.I.) for that study. In the pooled analysis, the diamond symbol represents the pooled estimate, with the right and left points of the diamond indicating the 95% C.I. for the overall analysis

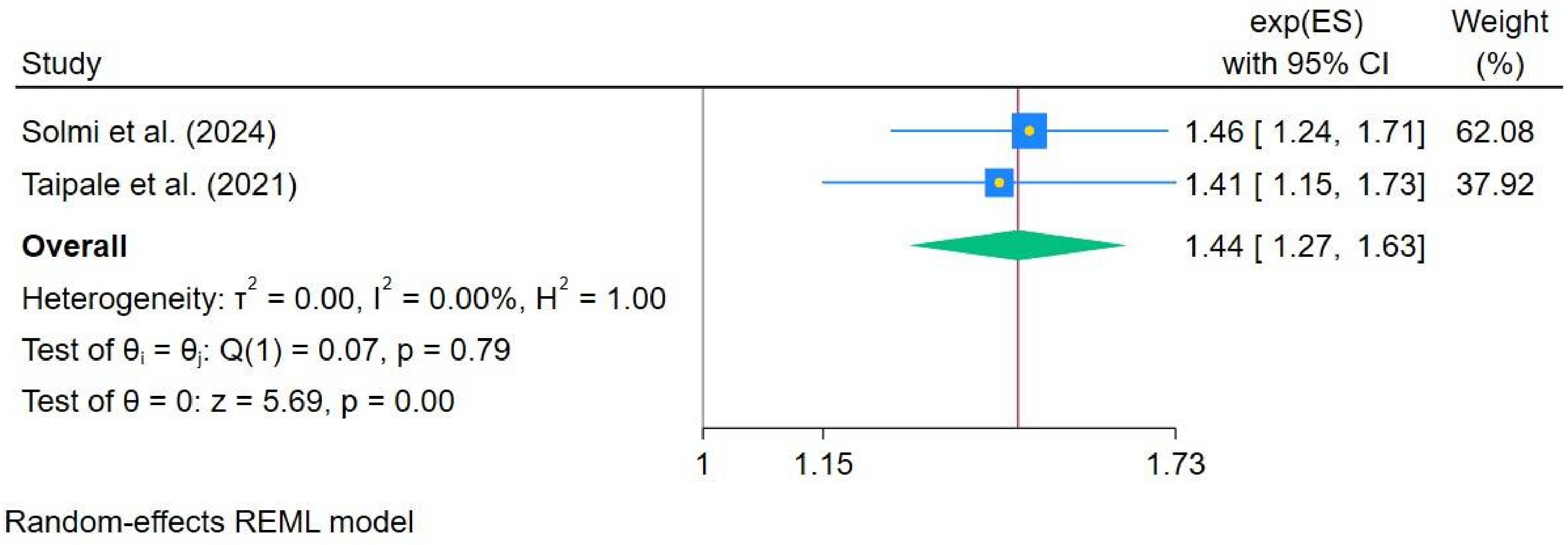

Figure S9. Cumulative Dispensed DDD – Any AP – >2000 DDD

The area of each square is proportional to the sample size of the respective study, with horizontal lines through the squares representing the 95% confidence interval (C.I.) for that study. In the pooled analysis, the diamond symbol represents the pooled estimate, with the right and left points of the diamond indicating the 95% C.I. for the overall analysis

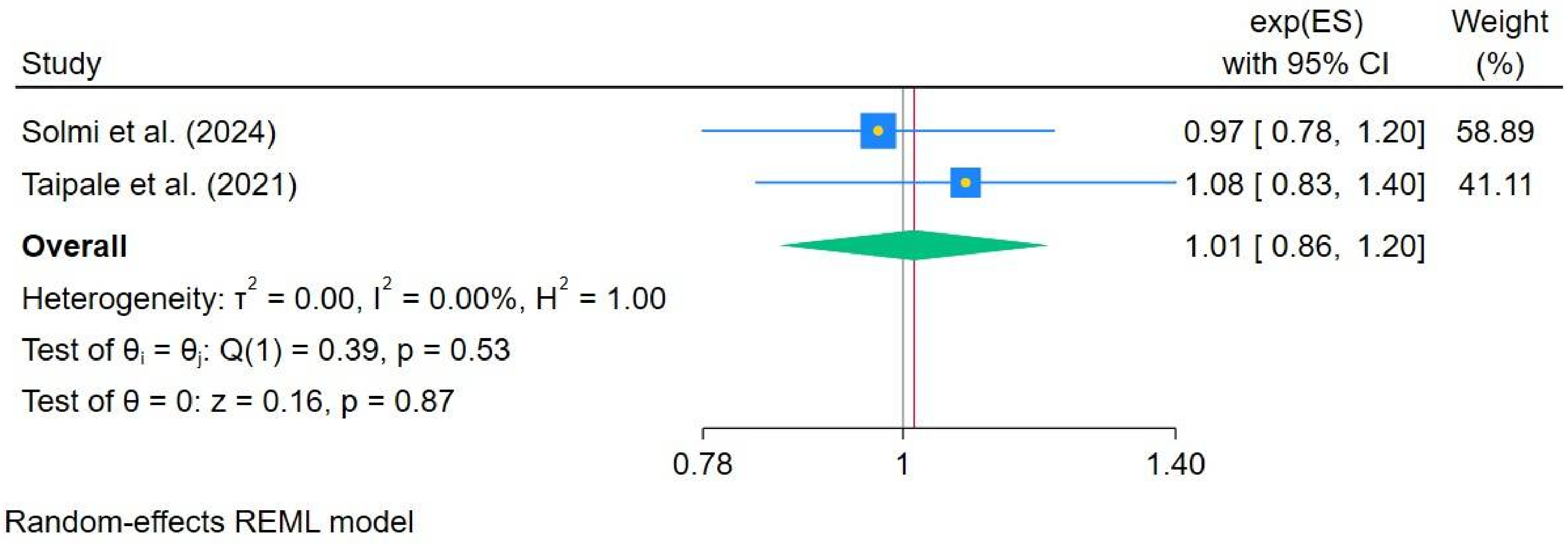

Figure S10. Cumulative Dispensed DDD – PIAP – 500-999 DDD

The area of each square is proportional to the sample size of the respective study, with horizontal lines through the squares representing the 95% confidence interval (C.I.) for that study. In the pooled analysis, the diamond symbol represents the pooled estimate, with the right and left points of the diamond indicating the 95% C.I. for the overall analysis

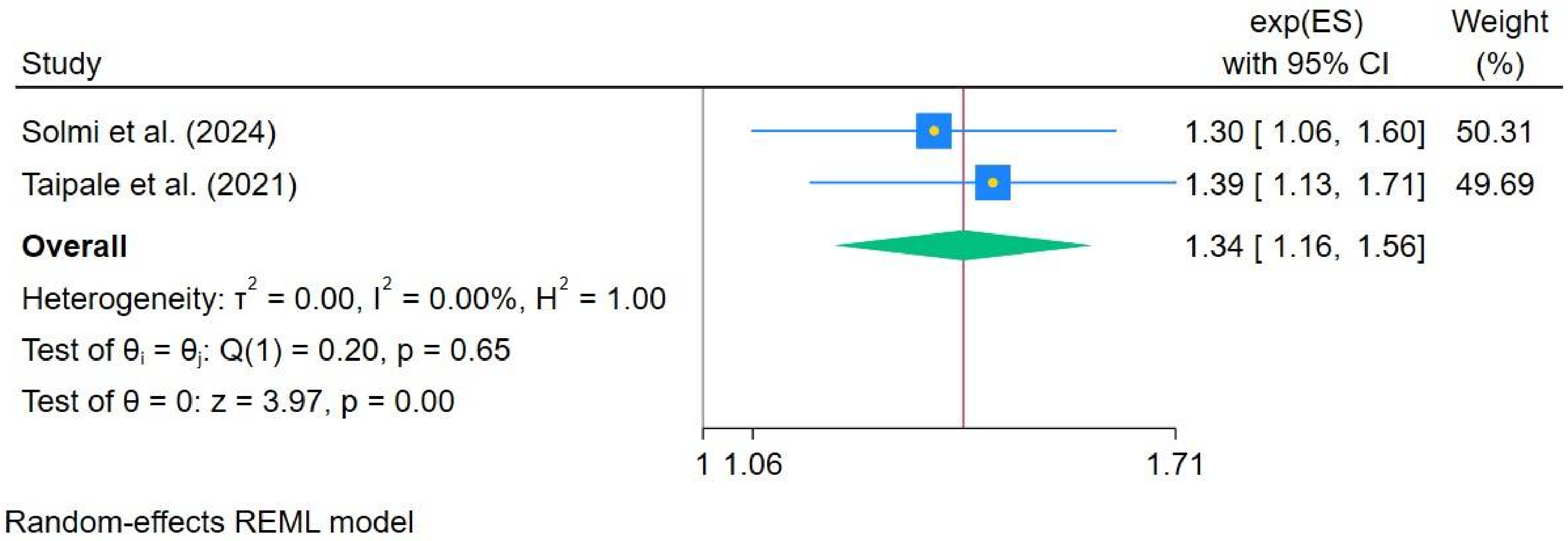

Figure S11. Cumulative Dispensed DDD – PIAP – 1000-1999 DDD

The area of each square is proportional to the sample size of the respective study, with horizontal lines through the squares representing the 95% confidence interval (C.I.) for that study. In the pooled analysis, the diamond symbol represents the pooled estimate, with the right and left points of the diamond indicating the 95% C.I. for the overall analysis

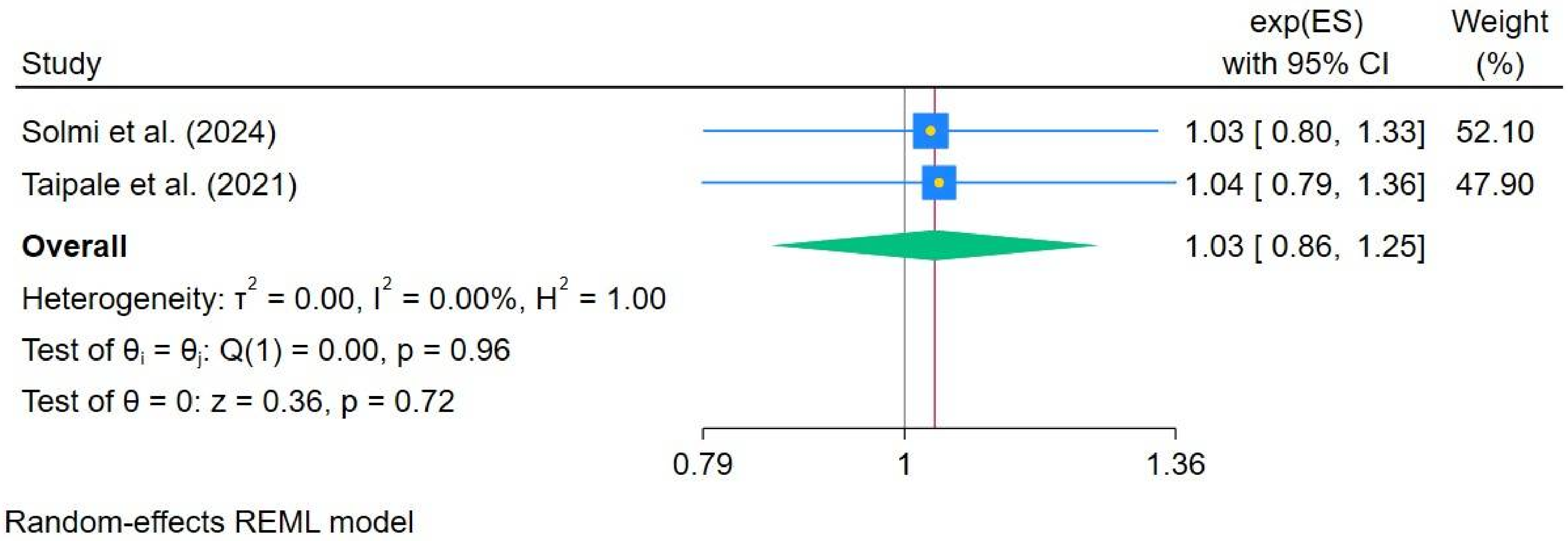

Figure S12. Cumulative Dispensed DDD − PIAP − >2000 DDD

The area of each square is proportional to the sample size of the respective study, with horizontal lines through the squares representing the 95% confidence interval (C.I.) for that study. In the pooled analysis, the diamond symbol represents the pooled estimate, with the right and left points of the diamond indicating the 95% C.I. for the overall analysis

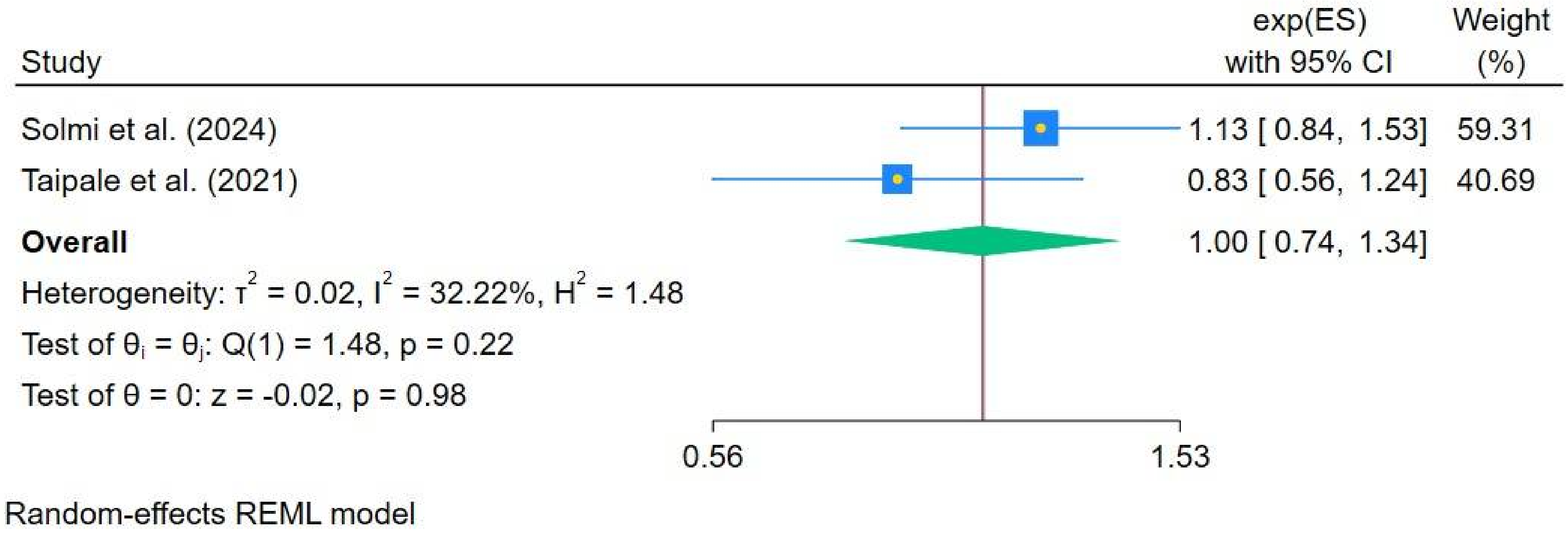

Figure S13. Cumulative Dispensed DDD – PSAP – 500-999 DDD

The area of each square is proportional to the sample size of the respective study, with horizontal lines through the squares representing the 95% confidence interval (C.I.) for that study. In the pooled analysis, the diamond symbol represents the pooled estimate, with the right and left points of the diamond indicating the 95% C.I. for the overall analysis

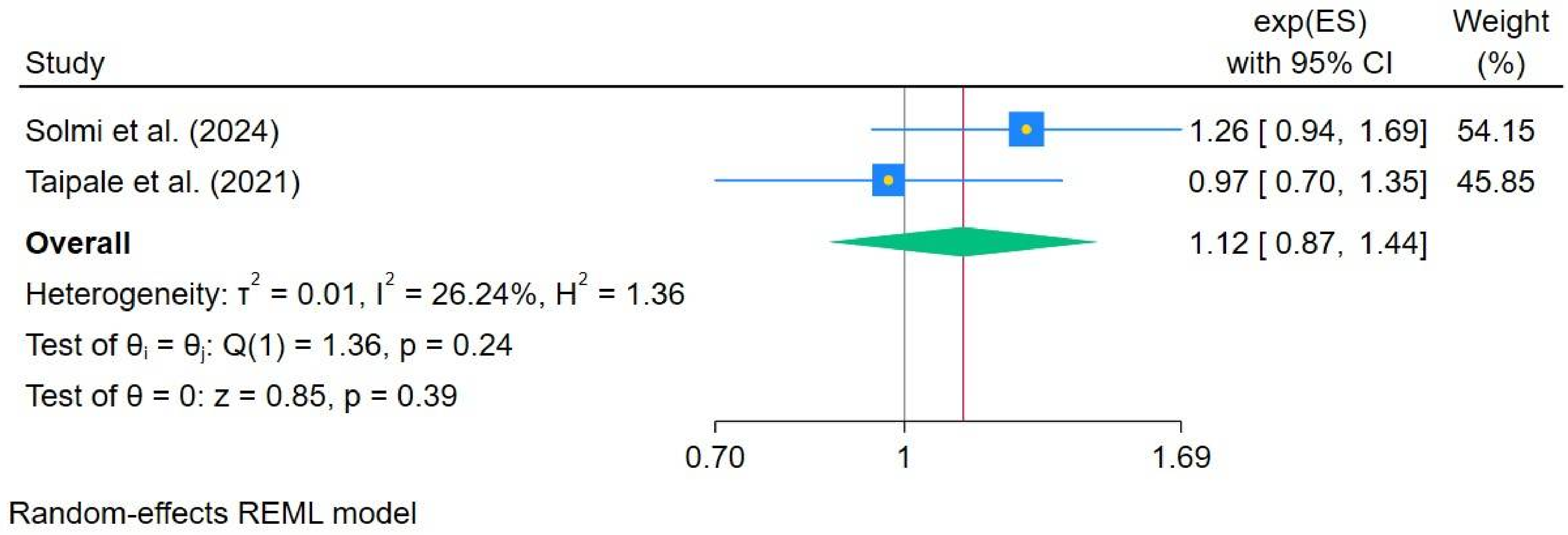

Figure S14. Cumulative Dispensed DDD – PSAP – 1000-1999 DDD

The area of each square is proportional to the sample size of the respective study, with horizontal lines through the squares representing the 95% confidence interval (C.I.) for that study. In the pooled analysis, the diamond symbol represents the pooled estimate, with the right and left points of the diamond indicating the 95% C.I. for the overall analysis

SPARING >2000

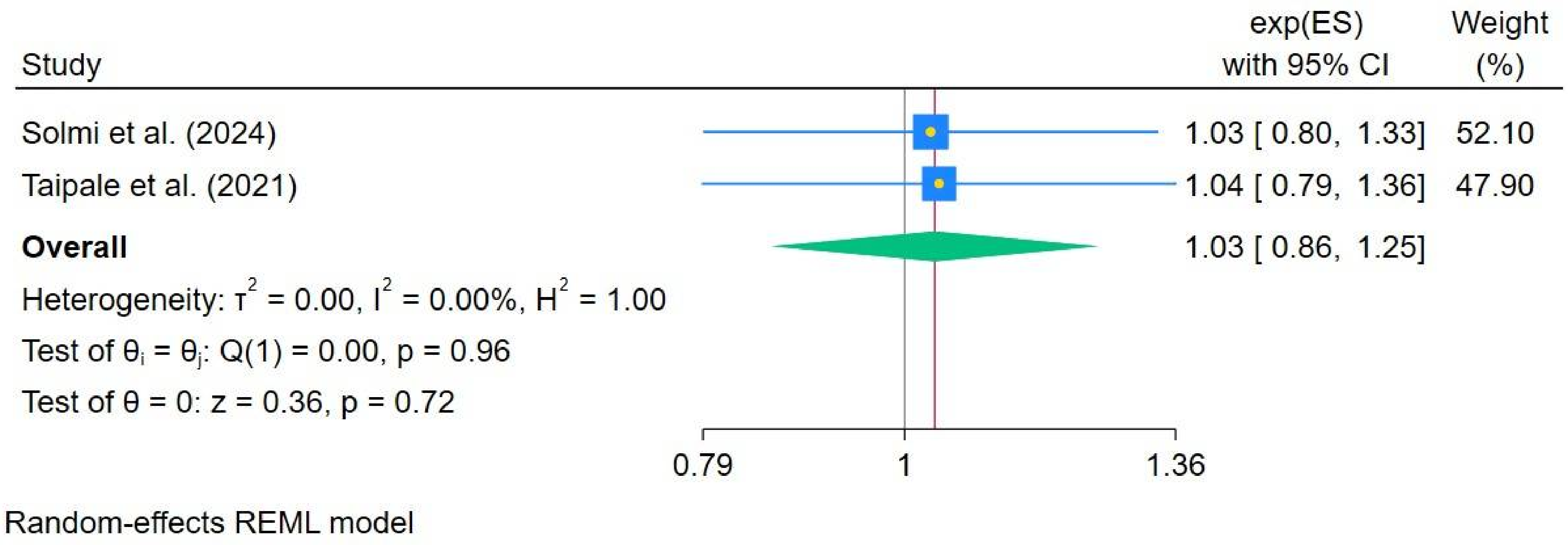

Figure S15. Cumulative Dispensed DDD – PSAP – >2000 DDD

The area of each square is proportional to the sample size of the respective study, with horizontal lines through the squares representing the 95% confidence interval (C.I.) for that study. In the pooled analysis, the diamond symbol represents the pooled estimate, with the right and left points of the diamond indicating the 95% C.I. for the overall analysis

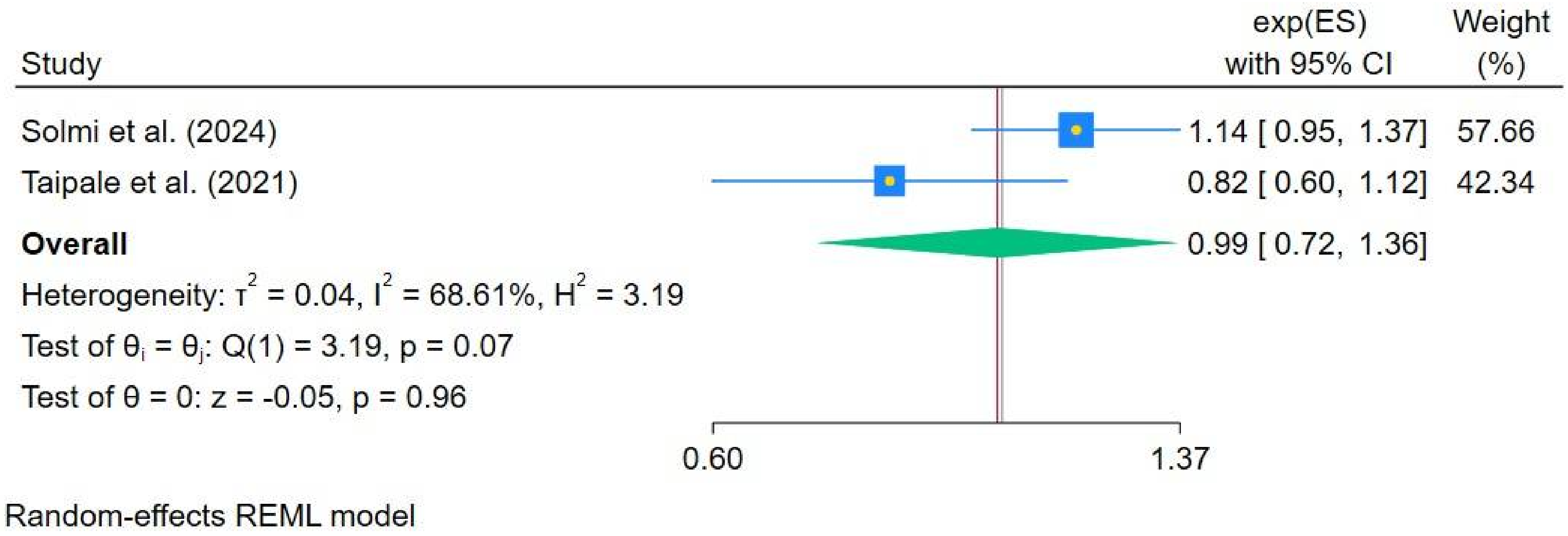

Figure S16. Ductal histotype – 1-5 years of use

The area of each square is proportional to the sample size of the respective study, with horizontal lines through the squares representing the 95% confidence interval (C.I.) for that study. In the pooled analysis, the diamond symbol represents the pooled estimate, with the right and left points of the diamond indicating the 95% C.I. for the overall analysis

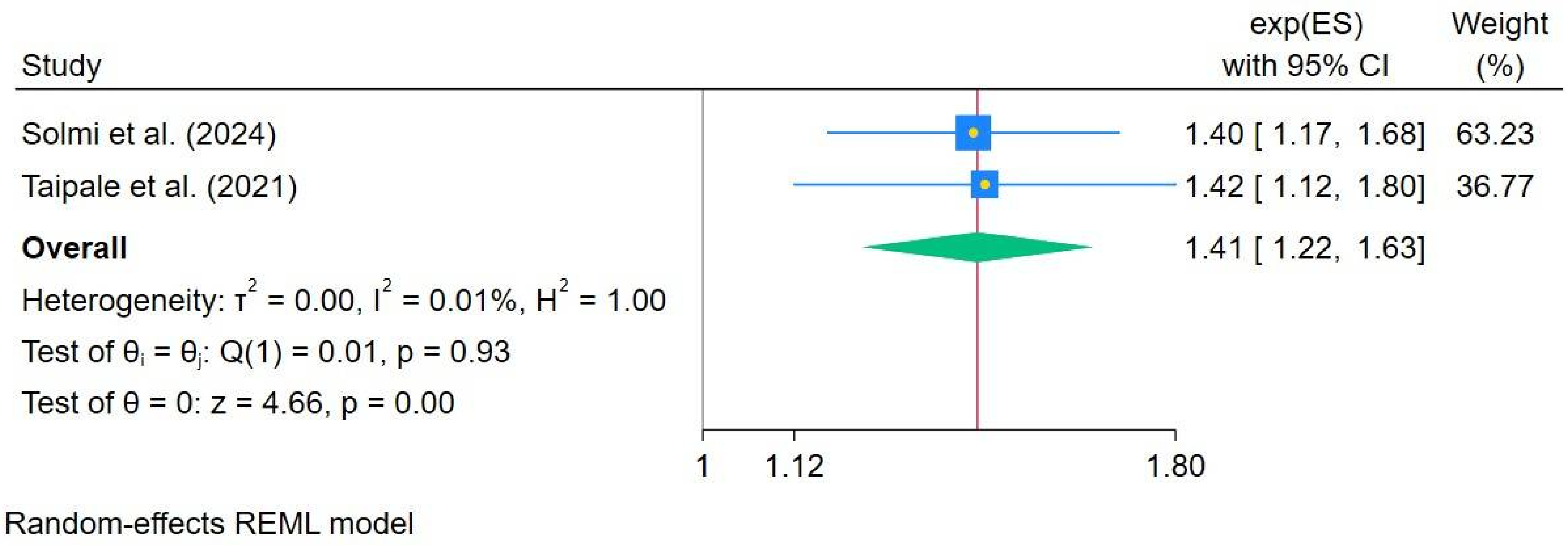

Figure S17. Ductal histotype – >5 years of use

The area of each square is proportional to the sample size of the respective study, with horizontal lines through the squares representing the 95% confidence interval (C.I.) for that study. In the pooled analysis, the diamond symbol represents the pooled estimate, with the right and left points of the diamond indicating the 95% C.I. for the overall analysis

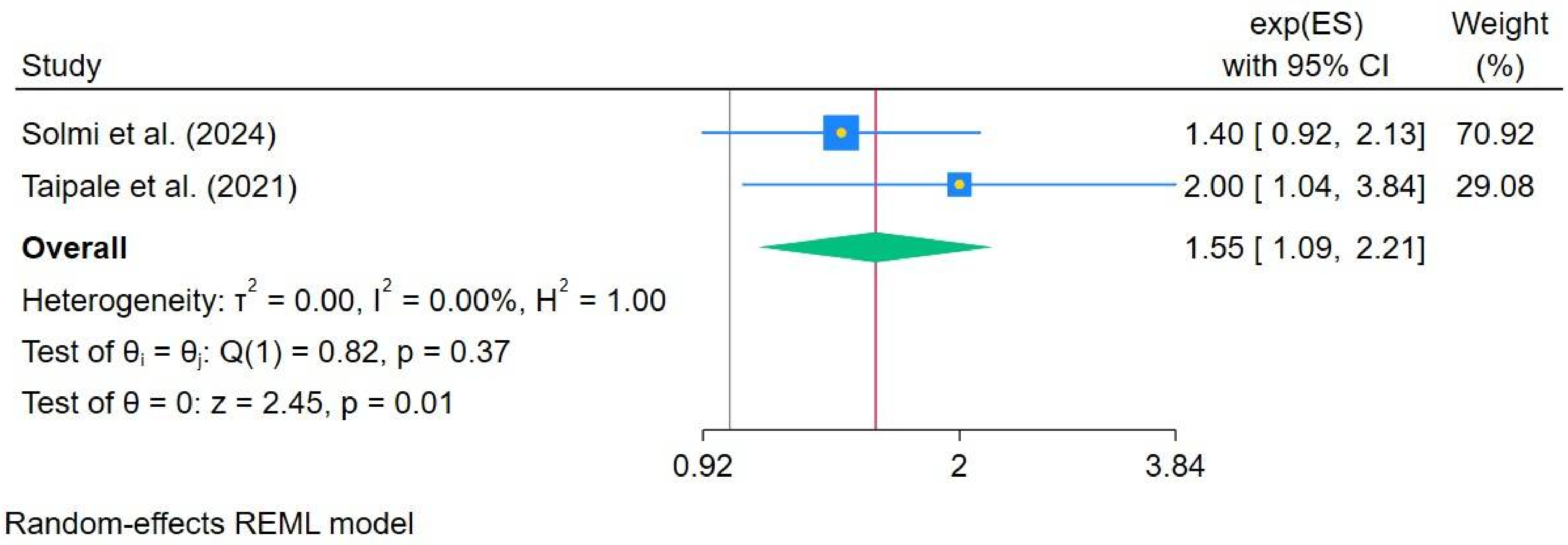

Figure S18. Lobular histotype – 1-5 years of use

The area of each square is proportional to the sample size of the respective study, with horizontal lines through the squares representing the 95% confidence interval (C.I.) for that study. In the pooled analysis, the diamond symbol represents the pooled estimate, with the right and left points of the diamond indicating the 95% C.I. for the overall analysis

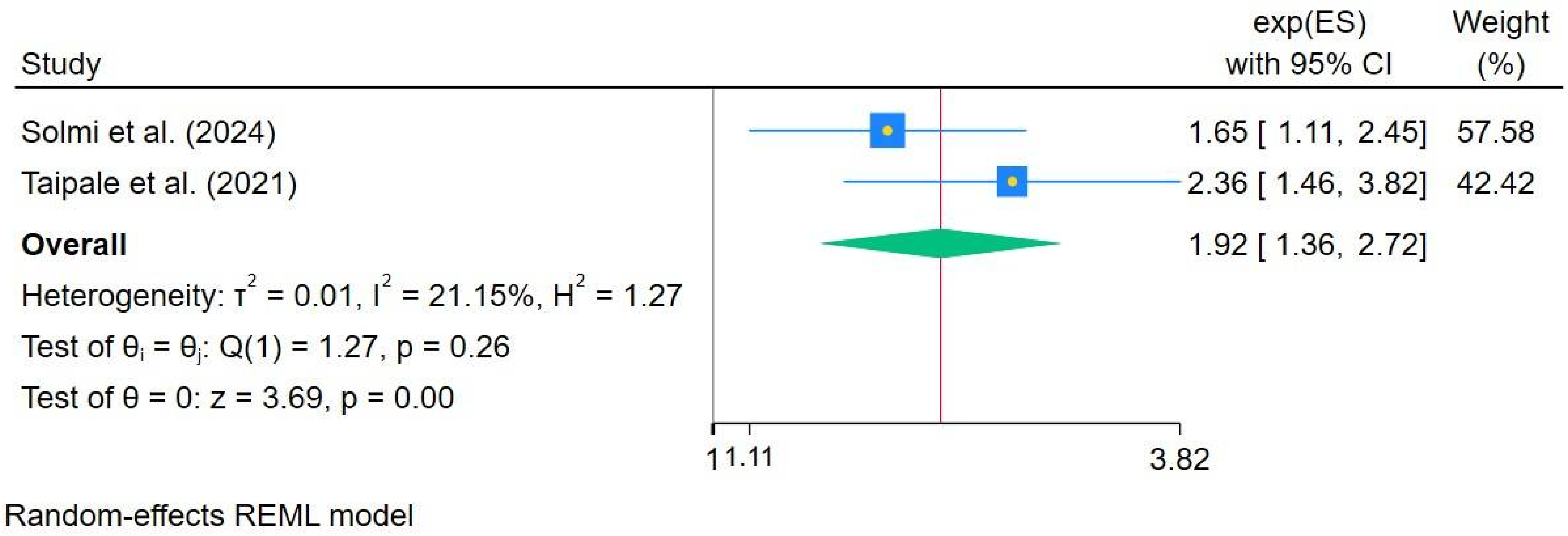

Figure S19. Lobular histotype – >5 years of use

The area of each square is proportional to the sample size of the respective study, with horizontal lines through the squares representing the 95% confidence interval (C.I.) for that study. In the pooled analysis, the diamond symbol represents the pooled estimate, with the right and left points of the diamond indicating the 95% C.I. for the overall analysis

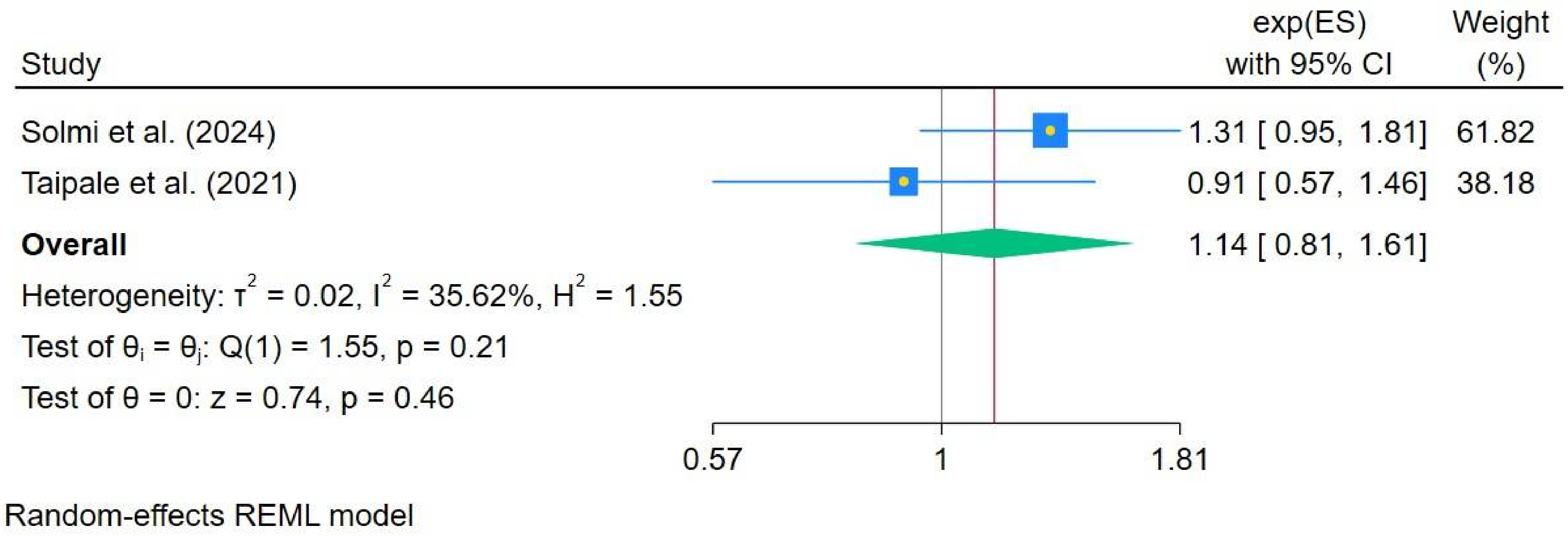

Figure S20. Age<55 years and use 1-5 years of use

The area of each square is proportional to the sample size of the respective study, with horizontal lines through the squares representing the 95% confidence interval (C.I.) for that study. In the pooled analysis, the diamond symbol represents the pooled estimate, with the right and left points of the diamond indicating the 95% C.I. for the overall analysis

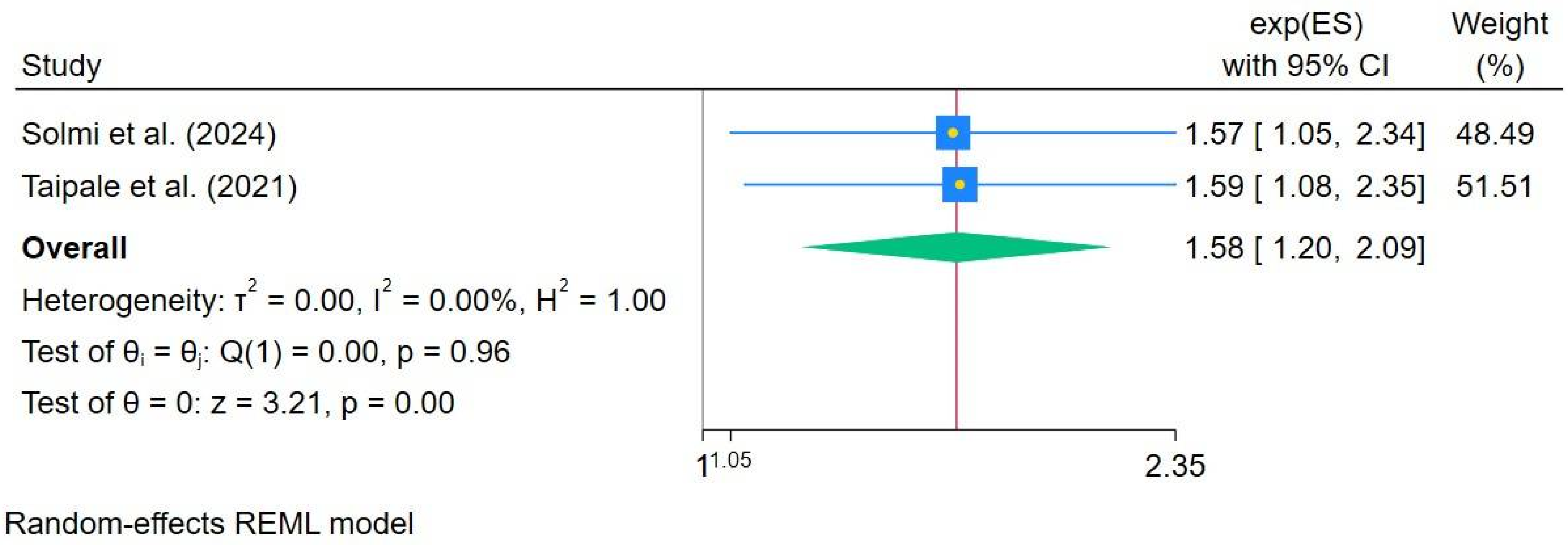

Figure S21. Age<55 years and use >5 years of use

The area of each square is proportional to the sample size of the respective study, with horizontal lines through the squares representing the 95% confidence interval (C.I.) for that study. In the pooled analysis, the diamond symbol represents the pooled estimate, with the right and left points of the diamond indicating the 95% C.I. for the overall analysis

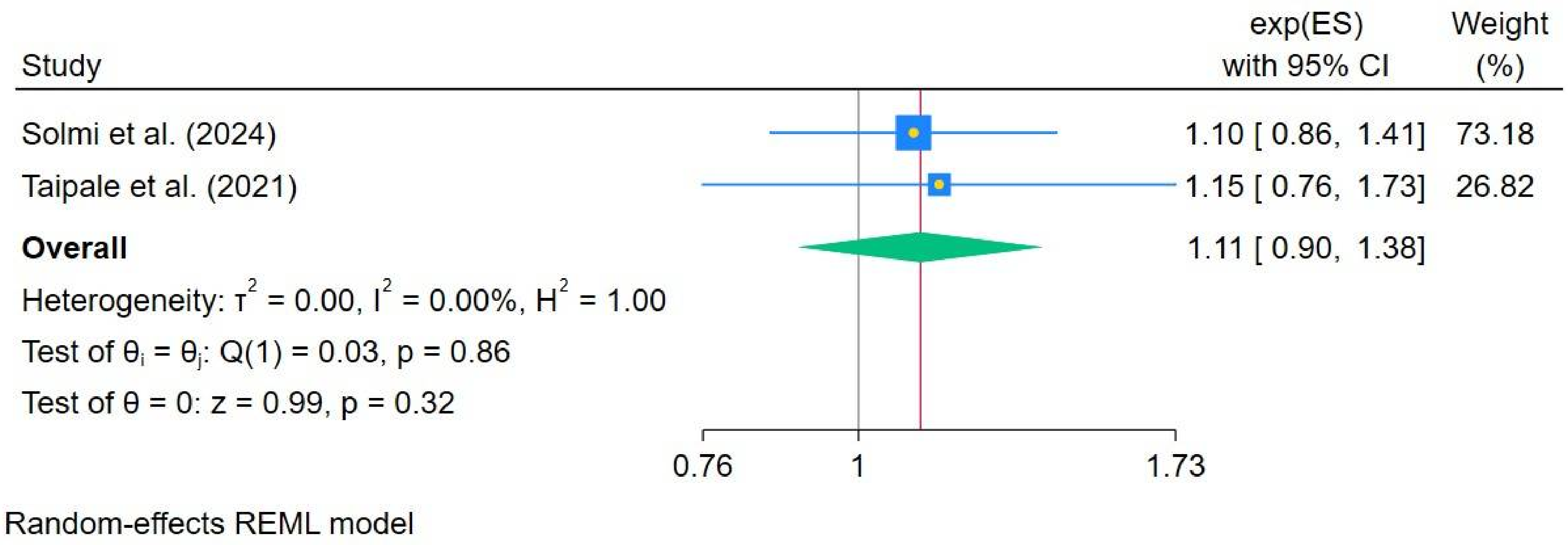

Figure S22. Age 55-69 years and use 1-5 years of use

The area of each square is proportional to the sample size of the respective study, with horizontal lines through the squares representing the 95% confidence interval (C.I.) for that study. In the pooled analysis, the diamond symbol represents the pooled estimate, with the right and left points of the diamond indicating the 95% C.I. for the overall analysis

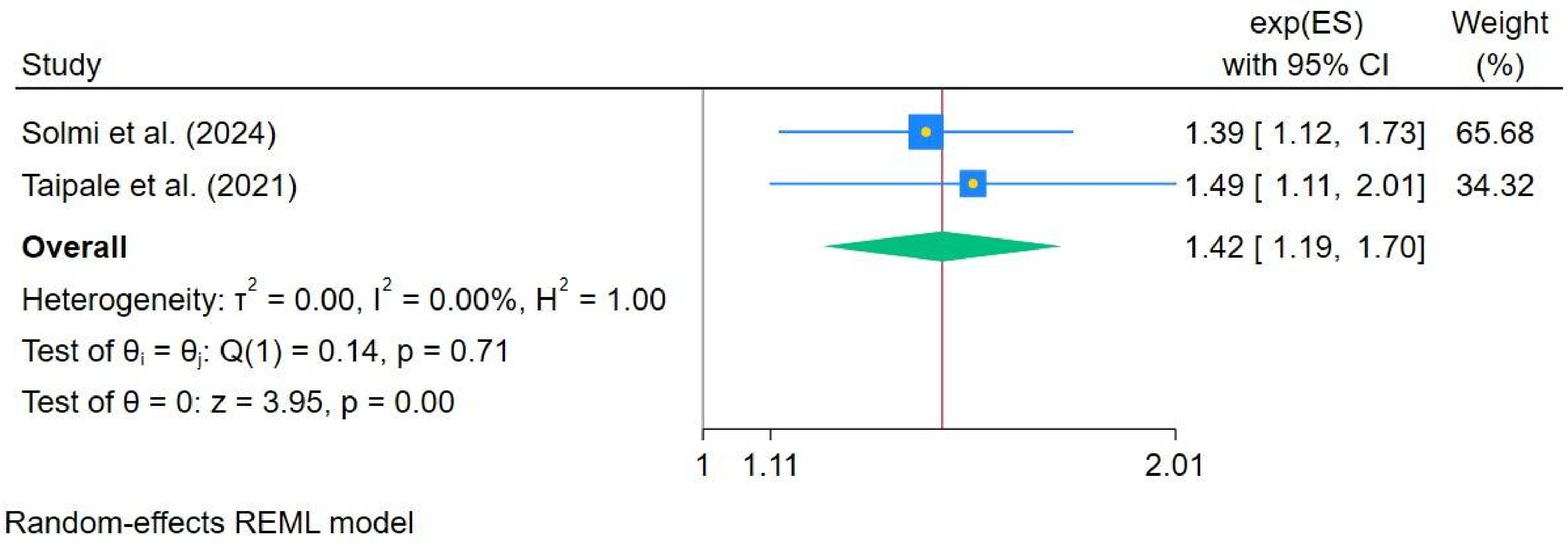

Figure S23. Age 55-69 years and use >5 years of use

The area of each square is proportional to the sample size of the respective study, with horizontal lines through the squares representing the 95% confidence interval (C.I.) for that study. In the pooled analysis, the diamond symbol represents the pooled estimate, with the right and left points of the diamond indicating the 95% C.I. for the overall analysis

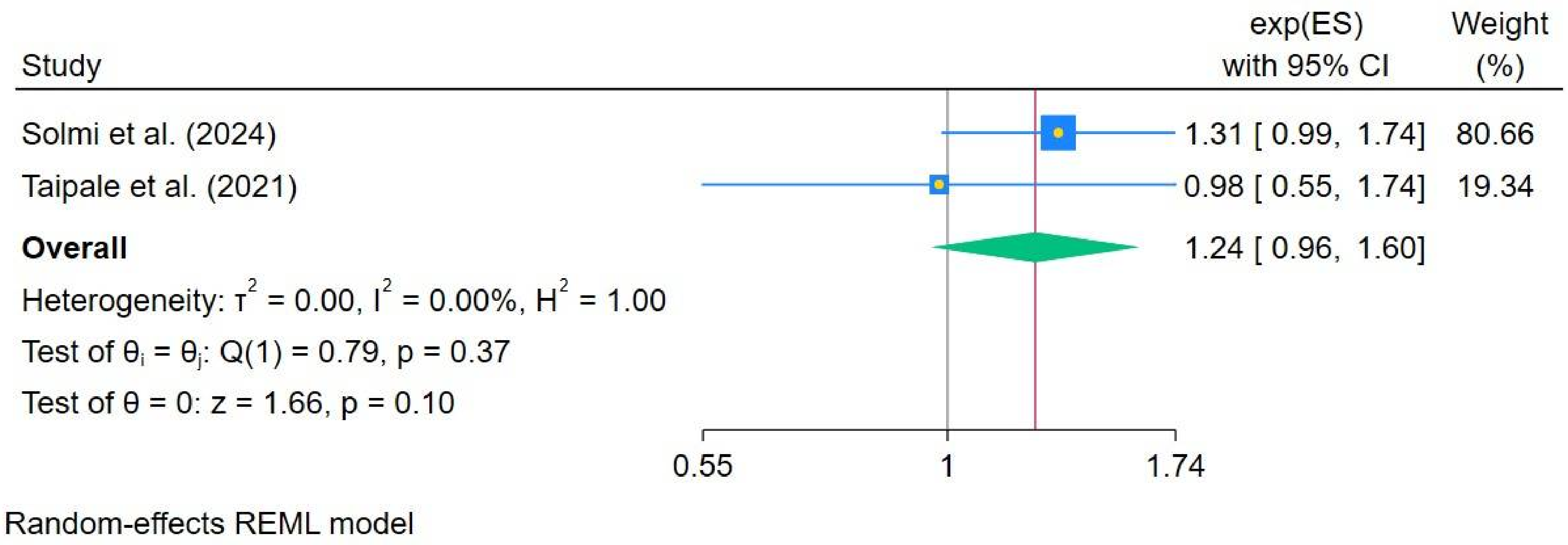

Figure S24. Age >70 and use 1-5 years of use

The area of each square is proportional to the sample size of the respective study, with horizontal lines through the squares representing the 95% confidence interval (C.I.) for that study. In the pooled analysis, the diamond symbol represents the pooled estimate, with the right and left points of the diamond indicating the 95% C.I. for the overall analysis

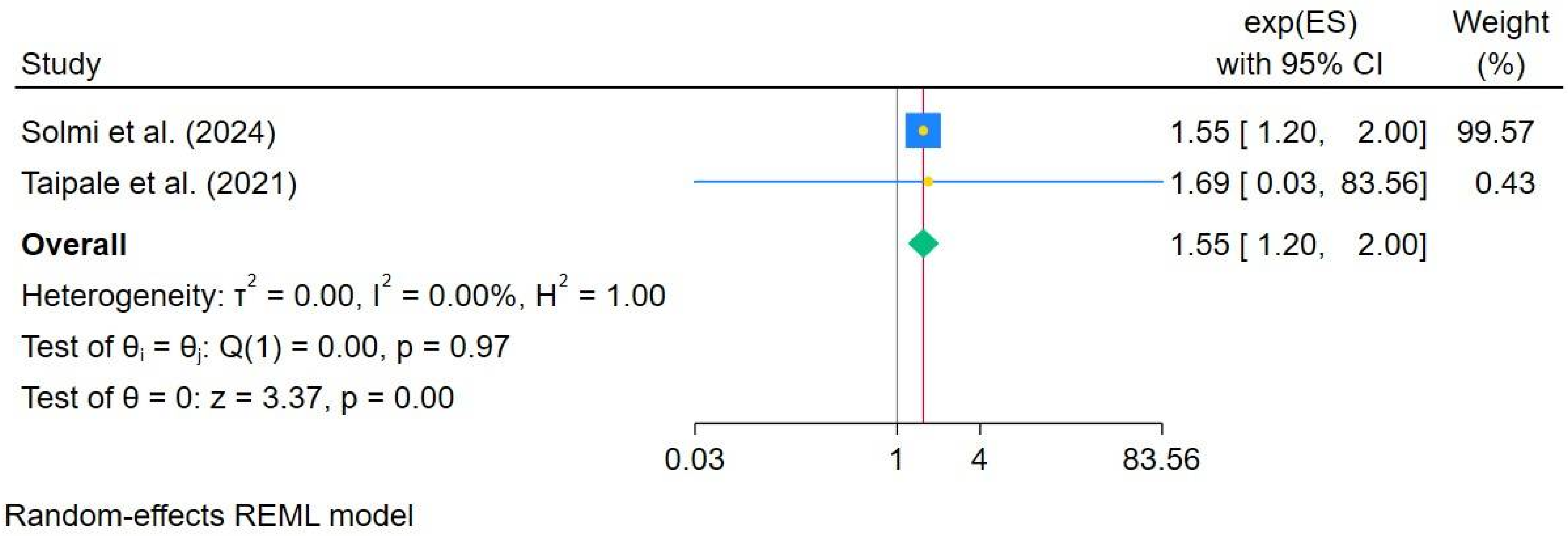

Figure S25. Age >70 and use >5 years of use

The area of each square is proportional to the sample size of the respective study, with horizontal lines through the squares representing the 95% confidence interval (C.I.) for that study. In the pooled analysis, the diamond symbol represents the pooled estimate, with the right and left points of the diamond indicating the 95% C.I. for the overall analysis
